# Examining the Effector Mechanisms of the Feishu Acupoint (BL13) in the Treatment of Pneumonia Based on Systematic Acupuncture and Moxibustion Research

**DOI:** 10.1155/2021/5578104

**Published:** 2021-07-05

**Authors:** Yan Xu, Jiali Cai, Weibin Li, Jingkun Miao, Yan Mei, Xinna Wang, Hanying Xu, Qixiong Chen, Fang Liu, Hongtao Cui

**Affiliations:** ^1^Changchun University of Chinese Medicine, Changchun 130117, China; ^2^Chongqing Hospital of Traditional Chinese Medicine, Chongqing 400021, China; ^3^Newborn Screening Center, Chongqing Health Center for Women and Children, Chongqing 401332, China; ^4^Chongqing Medical University, Chongqing 400016, China

## Abstract

**Background:**

Pneumonia is a serious global health problem. In traditional Chinese medicine, acupuncture or moxibustion is used to directly stimulate select acupoints on the surface of the human body and produce physical stimulation to further stimulate regulatory functions in the body, strengthening bodily resistance, eliminating disease, and adjusting the viscera. However, this Chinese medicine knowledge does not include the specific mechanisms of action or targets of acupoints. Therefore, an in-depth research is needed.

**Methods:**

An acupoint-element database was constructed, and the target elements of the Feishu point were screened. The UniProt-Swiss-Prot sublibrary was used to obtain correct gene name information. The National Center for Biotechnology Information (NCBI) Gene Expression Omnibus (GEO) database and GEO2R were used to analyze differentially expressed genes in pneumonia. The STRING database was used to analyze interactions, construct a network of the Feishu point efficacy system in pneumonia, and elucidate the mechanisms of action.

**Results:**

The Feishu point comprises 34 elements in total. The protein interaction analysis has 38 nodes and 115 edges. The Feishu point efficacy system-pneumonia system network shows that cytokine signaling in the immune system, signaling by interleukins (ILs), IL-4 and IL-13 signaling, and the immune system may be related to immunity and inflammation. The Feishu point efficacy system regulating pneumonia showed that FCER2, IL4R, FASLG, TGFB1, IL6R, STAT6, IL1B, CASP3, IL5RA, IL2RB, MYD88, SQSTM1, IL12RB1, IFNGR1, ADAM17, and CDH1 are the main targets.

**Conclusion:**

From the perspective of systematic acupuncture and moxibustion, the Feishu point regulates cytokine signaling in the immune system, signaling by ILs, IL-4 and IL-13 signaling, and the immune system by targeting FCER2, IL4R, FASLG, TGFB1, IL6R, STAT6, IL1B, CASP3, IL5RA, IL2RB, MYD88, SQSTM1, IL12RB1, IFNGR1, ADAM17, and CDH1, thereby regulating pneumonia.

## 1. Background

The Feishu point (BL13) was first described in *Lingshu Backshu*. It belongs to the bladder meridian (BL) and is the Back-Shu point of the lung. This point is located 1.5 inches beside and below the spinous process of the third thoracic vertebra on the back. The indications for Feishu point stimulation include pulmonary symptoms such as cough, expectoration, wheezing, and shortness of breath. Acupoint massage [1], applicator therapy [2, 3], and iontophoresis [4] at the Feishu point can shorten the time to cough improvement and reduce cough symptom scores, inflammatory indicator levels, and adverse reaction rates in patients with pneumonia. Moxibustion at the Feishu point can regulate fatigue and muscle aches in patients with severe acute respiratory syndrome coronavirus 2 (SARS-CoV-2) [5]. The efficacy of the Feishu point in the treatment of lung diseases has been verified for thousands of clinical applications, but studies on its specific mechanisms of action are rare. Because of the modernization and globalization of traditional Chinese medicine, the mechanisms of action of the Feishu point must be addressed.

Investigations of the mechanisms of action of acupoints must first explain the nature of meridians and collaterals. However, controversy regarding the nature of meridians and collaterals remains. Some scholars working from the perspective of modern medicine consider meridians and collaterals to be complicated three-dimensional structures composed of nerves, blood vessels, muscles, tendons, and fasciae. Acupuncture and moxibustion stimulate acupoints to cause changes in liquid components, nerve conduction, and physical properties in local tissues, which result in the alleviation or cure of the disease condition in the human body [6, 7]. However, some scholars disagree and consider that the understanding of the nature of “meridians and collaterals” is inseparable from the specific lifestyle, cognitive orientation, and language framework of traditional Chinese medicine, namely, traditional Chinese medicine culture and theory. The evolution of modern medical explanations and research methods deviates from the original meaning of the original classics [8]. The present study considered that current explorations of the philosophical theory of traditional Chinese medicine and the development of science and technology cannot resolve the issue of the nature of meridians and collaterals. However, methods that indirectly elucidate the mechanisms of action of acupoints may be applied.

**Figure 1:**
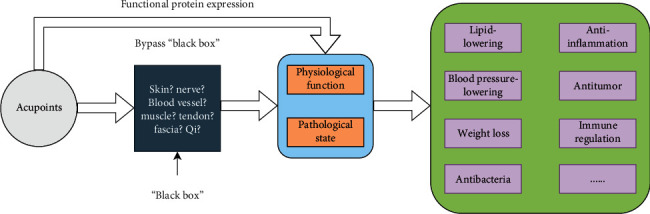
Road map of the mechanisms of action of acupoints.

Proteins are important constituents of all cells and tissues in the human body. All substances with physiological activities in the human body, such as amines, neurotransmitters, polypeptide hormones, antibodies, enzymes, nuclear proteins, and proteins that play “carrier” roles in cell membranes and in blood, are inseparable from proteins. Proteins play very important roles in the regulation of physiological functions and the maintenance of metabolism. Acupoint stimulation produces positive and negative regulatory effects in the body. The external presentation is disease improvement and recovery, and the internal presentation is an increase or decrease in protein levels. Therefore, the direct detection of changes in protein levels in the body bypasses the “black box” of the mechanisms of action of acupoints and indirectly elucidates these mechanisms (Figure 1).

Pneumonia is a serious global health problem [9], and it is the most common cause of death from infectious diseases worldwide, causing approximately 3.5 million deaths annually [10]. South Asia and Sub-Saharan Africa have the most severe pneumonia infections because of the economy and lack of medical care. Additionally, most deaths from pneumonia occur in children because of their unique physiological and pathological factors [11]. Acupuncture and moxibustion directly stimulate select acupoints on the surface of the human body and produce physical stimulation to further stimulate regulatory functions in the body, including the dredging of meridians and collaterals, the coordination of qi and blood, the strengthening of bodily resistance, the elimination of disease, and adjustment of the viscera. Because no special apparatus or equipment is required, acupuncture and moxibustion may be used as major methods for treating pneumonia in areas with scarce resources.

In summary, this study used systematic acupuncture and moxibustion to investigate the specific mechanisms of action of the Feishu point for regulating pneumonia. The results provide new ideas and methods for the globalization and moderation of acupuncture and moxibustion.

## 2. Methods

### 2.1. Construction of the Acupoint-Element Database

We performed a massive search of the literature in the China National Knowledge Infrastructure (CNKI), the Wanfang database, the Chongqing VIP Information network, PubMed, and Web of Science and constructed an acupoint-element (target) database for the 12 main meridians, conception vessels (CVs), and governor vessels (GVs) that included a total of 361 acupoints. Dedicated personnel rechecked, debugged, double-checked, and supplemented the results.

### 2.2. Retrieval of Target Elements of the Feishu Point

The Feishu point was retrieved from the acupoint-element (target) database, and a total of 34 target elements were obtained. Related information can be found on the website https://www.tcmmodel.com/feishu/. These target elements were entered into the UniProt database (https://www.uniprot.org/), one at a time for correction and conversion into gene names. UniProt is a nonredundant protein sequence database with the most complete sequence data and the most abundant annotated information worldwide. The more than 500,000 sequences in its Swiss-Prot sublibrary were manually reviewed and annotated. The correction databases used in this study used the Swiss-Prot sublibrary.

### 2.3. The Protein-Protein Interaction (PPI) Network and the Feishu Point Efficacy System Mechanism Network

The STRING database (https://string-db.org/) was used to search for interactions between proteins (gene names may be entered). It contains direct physical interactions between proteins and indirect functional correlations between proteins. The present study used the PPI network in STRING to evaluate elemental relationships. The median interaction score was 0.4.

The interaction score was the standard for determining the connections of the PPI network. The score was obtained via examination of the predictive performance using the public reference set (KEGG database), with the real relationship as the standard. The calculation formula was as follows:

(1) $$\begin{array}{c} Q=\log\left\{\frac{\left(N_{\text{together}}\cdot N_{\text{total}}\right)}{\left[\left(N_{{\text{alone}}_{1}}+1\right)\cdot \left(N_{{\text{alone}}_{2}}+1\right)\right]}\right\}. \end{array}$$

The diagram in this study concealed nodes that did not have interaction to ensure the reliability of the interactions in the PPI network.

### 2.4. Plotting the Network Diagram

The network of the interaction between the pneumonia efficacy system and pneumonia was plotted using Cytoscape (version 3.7.2). The degree value reflected the number of connections that a node in the network had with other nodes, which was the most intuitive parameter for determining the influence of a node. When the degree was larger, the influence was greater. Betweenness was the ratio of the closeness of nodes that passed through a certain node to the total closeness. When the betweenness was larger, the node was more important in the network. Closeness was the reciprocal of the average distance of the shortest paths between a node and other nodes in the network. Closeness considered the average length of the shortest paths between each node and other nodes. When a node was closer to other nodes, its closeness was higher.

### 2.5. Differentially Expressed Genes in Pneumonia

Differentially expressed genes were obtained from the National Center for Biotechnology Information (NCBI) Gene Expression Omnibus (GEO) database (https://www.ncbi.nlm.nih.gov/). The GEO is an online database that may be used to retrieve the gene expression data for any species. The GSE103119 data used in the present study contained the transcriptome data of 152 children with community-acquired pneumonia and 20 healthy children. The GEO2R online tool was used for analysis [12], and |LogFC| > 1 and *P* < 0.05 were used as the standards for screening differentially expressed genes. Children were chosen as subjects because children are the main victims of pneumonia.

### 2.6. Reactome Pathways

Reactome (https://reactome.org/) is a biological database of various reactions and biological pathways in the human body that has annotated more than 70% of human proteins. This study used the Reactome database for the functional analysis. The false discovery rate (FDR) was defined as the percentage of all discoveries that were false. When the FDR was smaller, the result was more reliable. The formula was as follows:

(2) $$\begin{array}{c} \text{FDR}=\frac{\text{FP}}{\left(\text{FP}+\text{TP}\right)}, \end{array}$$

where FP (false positive) indicated that the detection result of a negative experimental sample was positive and TP (true positive) indicated that the detection result of a positive experimental sample was positive.

## 3. Results

### 3.1. Identification of Target Elements in the Feishu Point Efficacy System

The targets were the focus of the construction of the Feishu point efficacy system and were considered the basic routes by which acupoints exert their effects. The targets of the Feishu point were obtained via a database search using the search term “Feishu” (Table 1).

### 3.2. Correction of Target Element Names in the Feishu Point Efficacy System

The target element names in the database were obtained from the literature search, and the nomenclature of targets in the literature varied slightly according to the reagent company. The target elements were entered into the UniProt database one at a time to standardize the nomenclature. All genes were uniformly expressed as gene names (Table 2).

### 3.3. Construction of the Structure and Relationship of the Feishu Point Efficacy System

**Figure 2:**
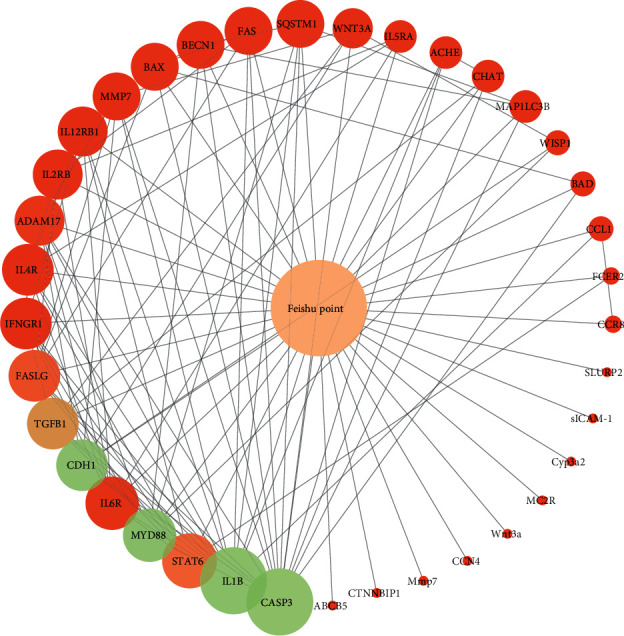
Relationship between the structure and efficacy of the Feishu point. The circle represents the degree, with larger circles corresponding to greater degrees. The color represents betweenness, with light green representing extensive betweenness and red representing limited betweenness.

The STRING website was used to perform a protein interaction analysis of all targets of lung acupoints. Then, the network of relationships among the elements was built, and a structure-relation network was constructed (Figure 2). There were 38 nodes and 115 edges in the network. To extract the key elements in the network, calculations were performed based on degree, betweenness, and closeness. Elements with the higher-than-average degree, betweenness, and closeness values were considered key elements in the network (Table 3).

### 3.4. Construction of the Feishu Point Efficacy System Network

To describe the Feishu point efficacy system network in detail, Reactome pathway enrichment was used to define the boundary of the system using the FDR parameter as the standard (Table 4).

### 3.5. Differentially Expressed Genes in Pneumonia

Differentially expressed genes in pneumonia were obtained from the GEO database. Differentially expressed genes were screened from healthy children and children with community-acquired pneumonia, and a total of 1074 genes were obtained (supplementary file (available here)).

### 3.6. Construction of the Feishu Point Efficacy System-Pneumonia System Network

**Figure 3:**
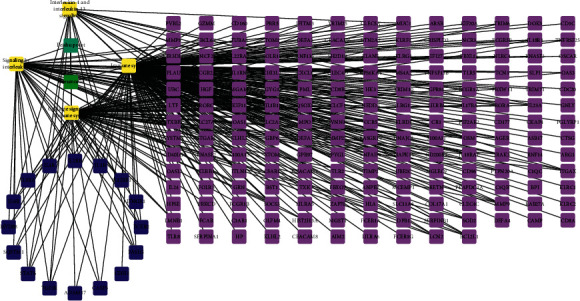
The Feishu point efficacy system-pneumonia system interaction network.

Systems do not exist in isolation. Similar to elements, systems also have relationships and structures. Therefore, the Feishu point efficacy system may also have a relationship with the pneumonia system. This relationship would be the key point of interaction between the two systems, and all physiological and biological functions would be based on this relationship. The construction of the Feishu efficacy system-pneumonia system network was equivalent to artificially determining the boundary of the Feishu point efficacy system so that only the parts of the Feishu point efficacy system that were relevant to pneumonia could be studied. After establishing the correspondence between the Feishu point efficacy system and the differentially expressed targets in pneumonia, the Feishu point efficacy system-pneumonia network relationship diagram was constructed (Figure 3). The Feishu point efficacy system had 4 pathways that could function in pneumonia, including cytokine signaling in the immune system, signaling by interleukins (ILs), IL-4 and IL-13 signaling, and the immune system. These 4 pathways were all associated with immunity and inflammation.

### 3.7. Mechanism of Action of by which the Feishu Point Efficacy System Regulates Pneumonia

**Figure 4:**
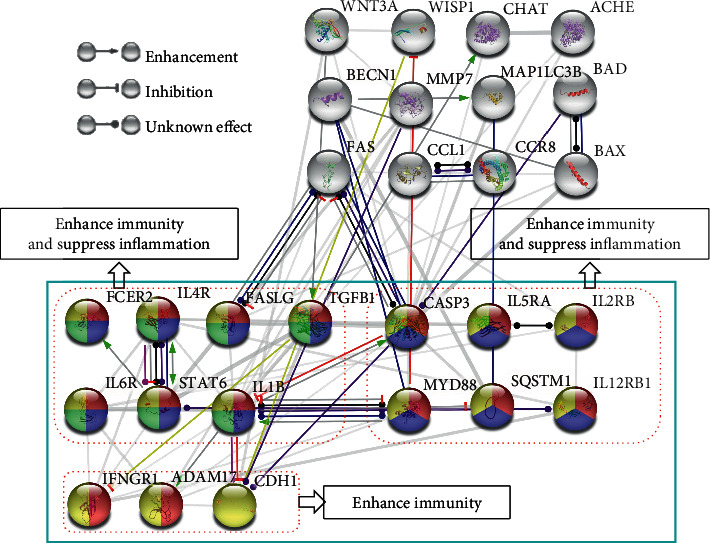
Diagram of the mechanisms of action of the Feishu point efficacy system in the treatment of pneumonia. Red indicates cytokine signaling in the immune system, blue indicates signaling by ILs, green indicates IL-4 and IL-13 signaling, yellow indicates the immune system, and white indicates other systems.

The roles of all of the proteins in the Feishu point efficacy system in the efficacy network were further examined. A median interaction score of 0.4 was used as the cutoff value for the plot shown in Figure 4. Sixteen proteins were involved in the mechanism by which the Feishu point efficacy system regulates pneumonia. FCER2, IL4R, FASLG, TGFB1, IL6R, STAT6, and IL1B were involved in the functions of all pathways, and CASP3, IL5RA, IL2RB, MYD88, SQSTM1, and IL12RB1 were involved in cytokine signaling in the immune system, IL signaling, and the immune system. IFNGR1 and ADAM17 were involved in cytokine signaling in the immune system and the immune system, and CDH1 was involved in the immune system.

## 4. Discussion

### 4.1. Theoretical Basis of Systematic Acupuncture and Moxibustion

**Figure 5:**
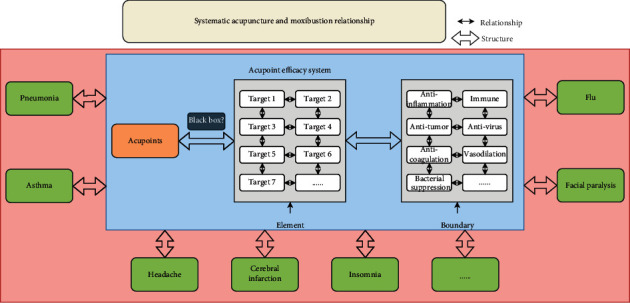
Systematic acupuncture and moxibustion research methods.

The extensive application of network pharmacology, systems pharmacology, and integrated pharmacology fields in recent years has promoted the development of molecular biology and pharmacology-related subjects that integrate systems science theory. These methods and technologies have focused on interactions between the body and drugs at the overall level and have promoted the preliminary establishment of a theory of systematic traditional Chinese medicine [32, 33]. Against the backdrop of systematic traditional Chinese medicine theory, the present study proposed a concept of systematic acupuncture and moxibustion that could affect the mechanisms of action of acupoints, meridians, and collaterals. This concept considered systematic acupuncture and moxibustion as a multidisciplinary subject of epistemology, a methodology that uses systems science thinking and methods to understand acupoints, meridians, and collaterals. This emerging discipline uses systems engineering technology to elucidate microscopic and macroscopic structures and relationships and can be applied to the science and art of acupoints, meridians, and collaterals. It is also an interdisciplinary subject that integrates acupuncture and moxibustion with systems science to unravel the self-similarity, self-organization, and self-adaptability of systems and elucidate the inheritance, additivity, and emergence of overall functions. The basic concept of systematic acupuncture and moxibustion includes elements and the relationships among elements, structures, boundaries, and functions. In systems science, a system is a whole that interacts with the external environment and has specific capabilities [34]. Elements are basic factors that constitute a system. Relationships refer to relationships between elements at the same level. Structure refers to the relationships between elements at different levels. The boundary is the boundary between the inside and outside of a system. Functions refer to the characteristics, behavior, performance, and functions of interactions between the system and the external environment [35]. Therefore, the use of data mining technology and biological network technology to confirm elements, structures, boundaries, and functions from the perspective of systematic acupuncture and moxibustion may systematically explain the mechanisms of action of acupoints, meridians, and collaterals (Figure 5).

### 4.2. Investigation of the Mechanisms of Action of the Feishu Point for the Regulation of Pneumonia

**Figure 6:**
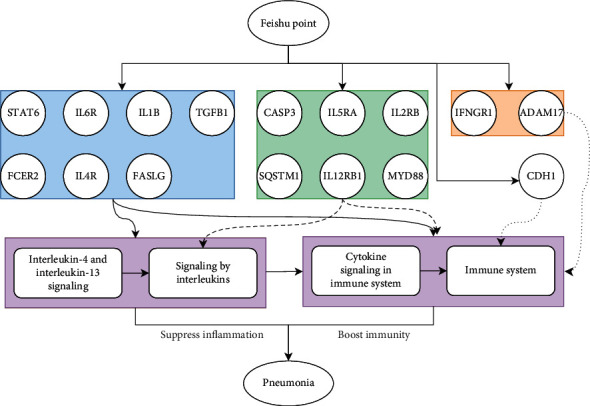
Mechanisms of action of the Feishu point in the regulation of pneumonia.

The present study demonstrates that the Feishu point exerts therapeutic effects in pneumonia via cytokine signaling in the immune system, IL signaling, IL-4 and IL-13 signaling, and the immune system. Cytokines are peptide substances that are extensively present in the body. Cytokines are secreted by immune cells, such as macrophages, B lymphocytes, T lymphocytes, and mast cells, as well as endothelial cells, fibroblasts, and various stromal cells. Based on their biological functions, cytokines are divided into chemokines, interferons, ILs, lymphokines, and tumor necrosis factors (TNFs). Cytokines in body fluids or tissues regulate the development, differentiation, and function of immune cells at a lower level under normal conditions to maintain the balance of the cytokine network. Once the body encounters abnormalities, the balance between anti-inflammatory cytokines and proinflammatory cytokines is impaired, and large amounts of proinflammatory cytokines are produced. Cells and intracellular signaling pathways also change accordingly, resulting in inflammation and immune processes [36]. IL-4 is primarily produced by activated T helper 2 (Th2) cells and mast cells, and it plays a role in the production of Th2 cytokines and the immunoregulatory function of lymphocytes and macrophages. IL-4 blocks antibody-dependent cytotoxicity, inhibits the production of IL-1*β*, TNF-*α*, prostaglandin E2, IL-6, IL-8, and nitric oxide, and induces B cells to produce IgG and IgE [37]. IL-13 also downregulates the synthesis of TNF-*α*, IL-1, and IL-6 and promotes IgE synthesis [38]. Overall, as important cytokines, IL-4 and IL-13 play important roles in mitigating the development of inflammatory responses and increasing immunity. At the gene level, this study demonstrates that the Feishu point regulates cytokine signaling in the immune system, signaling by ILs, IL-4 and IL-13 signaling, and the immune system via FCER2, IL4R, FASLG, TGFB1, IL6R, STAT6, IL1B, CASP3, IL5RA, IL2RB, MYD88, SQSTM1, IL12RB1, IFNGR1, ADAM17, and CDH1 gene regulation to exert effects on the regulation of inflammation and immunity (Figure 6), thereby preliminarily elucidating the mechanisms of action of the Feishu point in treating pneumonia.

Our research has some clinical significance. It differs from previous clinical studies that focused on different methods, such as application [39], cupping [40], and acupoint injection [41] to stimulate the Feishu acupoint and achieve the treatment goal. Our research starts with the physiological and pathological mechanisms that occur after acupoints are stimulated and adds specific biological evidence supporting their clinical effectiveness. Furthermore, unlike the previously described single mechanism [42, 43], we present a more comprehensive description of the mechanism through which lung acupoints regulate pneumonia.

### 4.3. Feishu Point Treatment System

**Figure 7:**
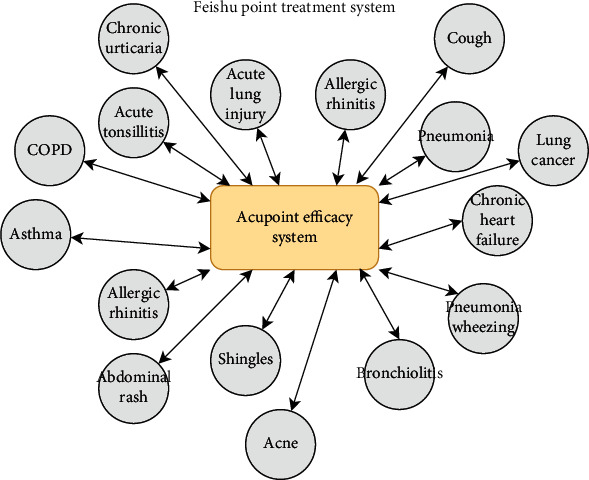
The Feishu point treatment system.

The Feishu point is a Back-Shu point on the bladder meridian (BL). It is located 1.5 inches beside and below the spinous process of the third thoracic vertebra and corresponds to the lung. The Feishu point is also the Back-Shu point of the lung. A complete review of the Feishu point treatment system based on existing studies showed very good efficacy of the Feishu point for the treatment of lung diseases, including cough [44], pneumonia with dyspnea and cough [45, 46], asthma [47], acute lung injury [48], allergic rhinitis [49], chronic obstructive pulmonary disease [50], lung cancer [51, 52], acute tonsillitis [49], and bronchiolitis [53]. The Feishu point efficacy system is also used to treat heart diseases, such as chronic heart failure [54], and skin diseases, such as chronic urticaria [55], shingles [56], abdominal urticaria [57], and acne [58]. To specifically elucidate the Feishu point efficacy system, it was considered as a whole according to systematic acupuncture and moxibustion theory and the evidence presented above [44–58] and plotted as a diagram (Figure 7). The diagram shows that the Feishu point treatment system is a complicated network that includes various diseases. This point plays a role in lung diseases, heart diseases and some skin diseases. This treatment system completely satisfies the theoretical bases of “all meridians and vessels converge in the lung to assist the heart in promoting blood circulation” and “the lung connects with the skin,” and it is a specific presentation of traditional Chinese medicine theory.

In summary, the elucidation of the therapeutic effect of the Feishu point from an overall perspective via the construction of the Feishu point treatment system met the basic characteristics of traditional Chinese medicine theory and provided scientific evidence for studies on the elucidation of the systematic functions and modernization of acupoints.

## 5. Conclusion

1. From the perspective of systematic acupuncture and moxibustion, this research explored the specific mechanism by which the Feishu point regulates pneumonia and provided new ideas for the development of acupuncture and moxibustion.
2. The Feishu point regulates cytokine signaling in the immune system, signaling by ILs, IL-4, and IL-13 signaling, and the immune system by targeting FCER2, IL4R, FASLG, TGFB1, IL6R, STAT6, IL1B, CASP3, IL5RA, IL2RB, MYD88, SQSTM1, IL12RB1, IFNGR1, ADAM17, and CDH1, thereby regulating pneumonia.

## Figures and Tables

**Table 1:** Target elements of the Feishu point.

Acupoint	Targets
Feishu point	LC3-II [13, 14], *p*62 [13], IL-6 [13, 15], Bax [16], Fas [16], FasL [16], Bcl-2 [16], IL-1*β* [17, 18], E-cad [19], MyD88 [20], caspase 3 [20], p-gp [21, 22], IL-12 [23, 24], IL-4 [15, 23, 24], IgE [23], IL-2 [24, 25], IL-5 [24], IFN-*γ* [15, 26, 27], TGF*β*1 [25, 28], *β*-catenin [29], MMP-7 [29], WISP-1 [29], Wnt3a [29], Beclin-1 [14], ACTH [15], CYP3A [22], TNF-*α* [22], sICAM-1 [25], CCL1 [30], CCR8 [30], STAT6 [30], ChAT [31], AChE [31], and mAChRs [31]

**Table 2:** Correction of target elements of the Feishu point.

Acupoint	Gene name
Feishu point	MAP1LC3B, SQSTM1, IL6R, BAX, FAS, FASLG, BAD, IL1B, CDH1, MYD88, CASP3, ABCB5, IL12RB1, IL4R, FCER2, IL2RB, IL5RA, IFNGR1, TGFB1, CTNNBIP1, MMP7, CCN4, Wnt3a, BECN1, MC2R, Cyp3a2, ADAM17, sICAM-1, CCL1, CCR8, STAT6, CHAT, AChE, and SLURP2

**Table 3:** Key elements of the Feishu point efficacy system.

Gene name	Degree	Betweenness	Closeness
CASP3	17	0.07995257	0.64912281
IL1B	17	0.06253873	0.64912281
STAT6	10	0.01346287	0.578125
MYD88	9	0.03216609	0.56923077
IL6R	9	0.00638972	0.55223881
CDH1	8	0.03350434	0.56060606
TGFB1	8	0.01788336	0.56060606
FASLG	8	0.0114984	0.55223881
IFNGR1	8	0.00362446	0.56060606
IL4R	8	0.00296964	0.54411765
ADAM17	7	0.00740502	0.55223881
IL2RB	7	0.00467134	0.53623188
IL12RB1	7	9.26E-04	0.53623188
MMP7	6	0.00372635	0.4625
BAX	6	0.0025025	0.53623188
BECN1	6	0.00234163	0.54411765
FAS	6	0.00187688	0.53623188
SQSTM1	6	0.00146575	0.54411765

**Table 4:** Top 10 Reactome enrichment results for the Feishu point efficacy system.

Reactome ID	Reactome description	FDR	Matched element
HSA-1280215	Cytokine signaling in the immune system	7.18*E* − 12	ADAM17, CASP3, FASLG, FCER2, IFNGR1, IL12RB1, IL1B, IL2RB, IL4R, IL5RA, IL6R, MYD88, SQSTM1, STAT6, and TGFB1
HSA-449147	Signaling by interleukins	1.31*E* − 11	CASP3, FASLG, FCER2, IL12RB1, IL1B, IL2RB, IL4R, IL5RA, IL6R, MYD88, SQSTM1, STAT6, and TGFB1
HSA-162582	Signal transduction	1.23*E* − 08	ADAM17, BAD, BAX, CASP3, CCL1, CCR8, CDH1, CTNNBIP1, FAS, FASLG, FCER2, IL2RB, IL5RA, IL6R, MC2R, MYD88, SQSTM1, STAT6, TGFB1, and WNT3A
HSA-6785807	Interleukin-4 and Interleukin-13 signaling	3.34*E* − 08	FASLG, FCER2, IL1B, IL4R, IL6R, STAT6, and TGFB1
HSA-73887	Death receptor signaling	1.62*E* − 07	ADAM17, BAD, CASP3, FAS, FASLG, MYD88, and SQSTM1
HSA-168256	Immune system	4.40*E* − 07	ADAM17, CASP3, CDH1, FASLG, FCER2, IFNGR1, IL12RB1, IL1B, IL2RB, IL4R, IL5RA, IL6R, MYD88, SQSTM1, STAT6, and TGFB1
HSA-109581	Apoptosis	9.96*E* − 06	BAD, BAX, CASP3, CDH1, FAS, and FASLG
HSA-5357801	Programmed cell death	9.96*E* − 06	BAD, BAX, CASP3, CDH1, FAS, and FASLG
HSA-193704	*p*75 NTR receptor-mediated signaling	1.68*E* − 05	ADAM17, BAD, CASP3, MYD88, and SQSTM1
HSA-5357769	Caspase activation via the extrinsic apoptotic signaling pathway	0.00032	CASP3, FAS, and FASLG

## Data Availability

The data used to support the findings of this study are included within the article.

## Supplementary Materials

Supplementary Materials 1074 differentially expressed genes in pneumonia.
