# Supplementary material for: Examining the Effector Mechanisms of the Feishu Acupoint (BL13) in the Treatment of Pneumonia Based on Systematic Acupuncture and Moxibustion Research
Source: Evid Based Complement Alternat Med. 2021 Jul 5;2021:5578104. doi: 10.1155/2021/5578104 (PMC8285197; doi:10.1155/2021/5578104)
Supplement: Supplementary Materials — 1074 differentially expressed genes in pneumonia. [file 5578104.f1.pdf]

ID	adj.P.Val	P.Value	logFC	Gene.symbo	Gene.title	Gene.ID
ILMN_1755[illegible]	4.35E-14	9.22E-19	-2.599154	SLC26A8	solute car	116369
ILMN_1763[illegible]	4.90E-13	2.54E-17	-1.467432	MTF1	metal regu	4520
ILMN_1708[illegible]	4.90E-13	3.12E-17	-1.170327	H2AFJ	H2A histor	55766
ILMN_1790[illegible]	1.45E-12	1.23E-16	-1.581387	CKAP4	cytoskelet	10970
ILMN_1785[illegible]	2.70E-12	2.86E-16	-3.031115	GPR84	G protein-	53831
ILMN_1697[illegible]	4.03E-12	5.13E-16	-1.571025	APOBR	apolipopro	55911
ILMN_2260[illegible]	4.94E-12	7.33E-16	-2.798784	NAIP	NLR family	4671
ILMN_1722[illegible]	5.79E-12	9.83E-16	-2.006691	TLR5	toll like	7100
ILMN_1768[illegible]	6.62E-12	1.26E-15	-1.44962	HIST2H2AC	histone cl	8338
ILMN_1892[illegible]	9.49E-12	2.01E-15	1.6762653	SNORD13	small nucl	692084
ILMN_1735[illegible]	9.91E-12	2.31E-15	-1.502876	KIF1B	kinesin fa	23095
ILMN_1678[illegible]	1.25E-11	3.17E-15	-1.622422	ZNF438	zinc finge	220929
ILMN_1762[illegible]	1.62E-11	4.45E-15	-2.819599	MCEMP1	mast cell	199675
ILMN_2126[illegible]	1.73E-11	5.46E-15	-1.6699	LMNB1	lamin B1	4001
ILMN_1674[illegible]	1.73E-11	6.13E-15	-2.749576	VNN1	vanin 1	8876
ILMN_2226[illegible]	1.73E-11	7.34E-15	-2.160501	INSL3	insulin li	3640
ILMN_2415[illegible]	1.73E-11	7.71E-15	-2.203234	CDK5RAP2	CDK5 regul	55755
ILMN_1812[illegible]	1.73E-11	7.65E-15	-3.856707	HP	haptoglobi	3240
ILMN_1711[illegible]	1.75E-11	8.55E-15	-2.612016	OPLAH	5-oxoproli	26873
ILMN_1743[illegible]	1.84E-11	9.37E-15	-1.83006	KIF1B	kinesin fa	23095
ILMN_2079[illegible]	2.11E-11	1.13E-14	1.6236873	KLRB1	killer cel	3820
ILMN_1765[illegible]	2.62E-11	1.57E-14	-1.322112	USB1	U6 snRNA b	79650
ILMN_1690[illegible]	3.35E-11	2.26E-14	-1.034845	ASAP1	ArfGAP wit	50807
ILMN_1775[illegible]	3.35E-11	2.28E-14	-1.270428	SLC2A3	solute car	6515
ILMN_1681[illegible]	4.21E-11	2.95E-14	-1.81391	AIM2	absent in	9447
ILMN_3242[illegible]	6.05E-11	4.49E-14	-1.405556	HIST2H2AA[illegible]	histone cl	723790
ILMN_1740[illegible]	7.87E-11	6.01E-14	-2.4067	TCN2	transcobal	6948
ILMN_1672[illegible]	1.17E-10	9.45E-14	-1.337643	ECE1	endothelin	1889
ILMN_1810[illegible]	1.24E-10	1.02E-13	-1.808405	DYSF	dysferlin	8291
ILMN_3238[illegible]	1.35E-10	1.15E-13	-2.153249	SMA4	glucuronic	11039
ILMN_1659[illegible]	1.55E-10	1.44E-13	-1.390704	HIST2H2AA[illegible]	histone cl	8337
ILMN_1708[illegible]	2.03E-10	1.94E-13	-1.635941	ADM	adrenomedu	133
ILMN_1803[illegible]	2.21E-10	2.16E-13	-1.341305	PHTF1	putative h	10745
ILMN_2388[illegible]	2.97E-10	3.02E-13	-2.126808	MAPK14	mitogen-ac	1432
ILMN_2230[illegible]	3.24E-10	3.36E-13	-1.73545	GYG1	glycogenin	2992
ILMN_1694[illegible]	3.39E-10	3.60E-13	-2.537783	ANXA3	annexin A3	306
ILMN_1707[illegible]	3.48E-10	3.80E-13	-1.429878	PLBD1	phospholip	79887
ILMN_1744[illegible]	3.48E-10	3.84E-13	-1.009572	ERO1A	endoplasmi	30001
ILMN_1769[illegible]	3.66E-10	4.11E-13	-1.03146	TBC1D2	TBC1 domai	55357
ILMN_1694[illegible]	3.92E-10	4.53E-13	-2.116297	LILRA6	leukocyte	79168
ILMN_1660[illegible]	3.92E-10	4.58E-13	-1.029502	LRPAP1	LDL recept	4043
ILMN_1658[illegible]	4.09E-10	4.85E-13	-1.006458	BAZ1A	bromodomai	11177
ILMN_1798[illegible]	4.24E-10	5.12E-13	-1.224538	SRPK1	SRSF prote	6732
ILMN_1660[illegible]	4.58E-10	5.73E-13	-1.835697	LIMK2	LIM domair	3985
ILMN_2176[illegible]	4.63E-10	5.89E-13	-2.309474	FCGR1A	Fc fragmer	2209
ILMN_1700[illegible]	5.01E-10	6.55E-13	-2.176361	KREMEN1	kringle cc	83999

ILMN_2394[illegible]	5.01E-10	6.58E-13	-2.428279	SLC26A8	solute car	116369
ILMN_2266[illegible]	5.19E-10	6.99E-13	-1.149937	LILRA5	leukocyte	353514
ILMN_1767[illegible]	5.19E-10	7.05E-13	-1.151234	RRP12	ribosomal	23223
ILMN_2144[illegible]	5.38E-10	7.42E-13	-1.402236	HIST2H2AA[illegible]	histone cl	8337
ILMN_1685[illegible]	5.54E-10	7.75E-13	-1.360103	B4GALT5	beta-1,4-g	9334
ILMN_1672[illegible]	7.52E-10	1.08E-12	-2.209115	SLC26A8	solute car	116369
ILMN_1667[illegible]	7.61E-10	1.13E-12	-1.044267	SQRDL	sulfide qu	58472
ILMN_1737[illegible]	8.12E-10	1.24E-12	-1.431564	BCL6	B-cell CLI	604
ILMN_1781[illegible]	1.02E-09	1.65E-12	-1.682174	MYBPC3	myosin bir	4607
ILMN_1664[illegible]	1.02E-09	1.60E-12	-2.888601	CACNA1E	calcium vc	777
ILMN_1702[illegible]	1.02E-09	1.67E-12	-1.263758	SEMA4A	semaphorir	64218
ILMN_1766[illegible]	1.02E-09	1.67E-12	-1.141134	SH3GLB1	SH3 domair	51100
ILMN_1685[illegible]	1.15E-09	1.95E-12	-1.774673	SLC22A4	solute car	6583
ILMN_2364[illegible]	1.34E-09	2.34E-12	-1.044413	GBA	glucosylce	2629
ILMN_1787[illegible]	1.69E-09	2.98E-12	-1.83754	C3AR1	complement	719
ILMN_1717[illegible]	1.77E-09	3.16E-12	-1.443859	AP5B1	adaptor re	91056
ILMN_1654[illegible]	1.99E-09	3.58E-12	-1.171532	HMGB2	high mobil	3148
ILMN_1651[illegible]	2.45E-09	4.53E-12	-1.289139	BASP1	brain abur	10409
ILMN_1701[illegible]	2.50E-09	4.73E-12	-1.865663	ALPL	alkaline p	249
ILMN_1766[illegible]	2.59E-09	5.05E-12	-1.242041	ABCA1	ATP bindir	19
ILMN_1755[illegible]	2.74E-09	5.47E-12	-1.102962	GBA	glucosylce	2629
ILMN_1747[illegible]	2.84E-09	5.79E-12	-2.4516	HIST2H2AB	histone cl	317772
ILMN_1813[illegible]	2.96E-09	6.09E-12	-1.218017	TRIM25	tripartite	7706
ILMN_1772[illegible]	3.01E-09	6.32E-12	-2.449319	KREMEN1	kringle cc	83999
ILMN_1812[illegible]	3.11E-09	6.66E-12	-3.204693	ARG1	arginase 1	383
ILMN_2092[illegible]	3.11E-09	6.67E-12	-1.713841	HPSE	heparanase	10855
ILMN_2050[illegible]	3.21E-09	7.03E-12	-1.653	SLC22A4	solute car	6583
ILMN_2261[illegible]	3.21E-09	7.09E-12	-2.055988	FCGR1B	Fc fragmer	2210
ILMN_1730[illegible]	3.24E-09	7.22E-12	-2.1165	FOLR3	folate rec	2352
ILMN_1726[illegible]	3.31E-09	7.50E-12	-1.770289	LILRA5	leukocyte	353514
ILMN_1669[illegible]	3.43E-09	7.94E-12	-1.367961	DRAM1	DNA damage	55332
ILMN_1733[illegible]	3.70E-09	8.64E-12	-1.604839	SULT1B1	sulfotrans	27284
ILMN_1747[illegible]	3.90E-09	9.27E-12	-1.467789	LTB4R	leukotrier	1241
ILMN_2268[illegible]	4.08E-09	1.00E-11	-1.091741	RRBP1	ribosome b	6238
ILMN_2113[illegible]	4.08E-09	1.00E-11	-1.30799	TOR1AIP2	torsin 1A	163590
ILMN_1670[illegible]	4.13E-09	1.03E-11	-1.67222	HK3	hexokinase	3101
ILMN_1811[illegible]	4.24E-09	1.06E-11	-1.021398	GRN	granulin	2896
ILMN_1766[illegible]	4.62E-09	1.17E-11	-1.149228	STOM	stomatin	2040
ILMN_1781[illegible]	4.66E-09	1.19E-11	-2.129556	SOCS3	suppressor	9021
ILMN_1760[illegible]	4.76E-09	1.22E-11	-2.296929	NAIP	NLR family	4671
ILMN_1679[illegible]	4.97E-09	1.30E-11	-1.263622	SERPINB1	serpin fam	1992
ILMN_1770[illegible]	5.40E-09	1.44E-11	-1.240628	DDAH2	dimethylar	23564
ILMN_1796[illegible]	5.88E-09	1.61E-11	-2.367294	MMP9	matrix met	4318
ILMN_1737[illegible]	6.35E-09	1.79E-11	-1.022588	N4BP2L2	NEDD4 binc	10443
ILMN_1764[illegible]	6.35E-09	1.78E-11	-1.940829	TECPR2	tectonin b	9895
ILMN_2367[illegible]	6.39E-09	1.81E-11	-1.52613	LIMK2	LIM domair	3985
ILMN_1727[illegible]	7.34E-09	2.10E-11	-1.364412	SSH1	slingshot	54434

ILMN_2123	1.13E-08	3.47E-11	-1.151217	FCER1G	Fc fragmer	2207
ILMN_1733	1.17E-08	3.61E-11	-1.050072	ARHGEF11	Rho guanir	9826
ILMN_2357	1.31E-08	4.15E-11	-1.737169	LILRA5	leukocyte	353514
ILMN_1798	1.34E-08	4.32E-11	-1.238225	UPP1	uridine pł	7378
ILMN_1766	1.34E-08	4.33E-11	-3.214835	ZDHHC19	zinc finge	131540
ILMN_1687	1.37E-08	4.45E-11	-1.728375	LIMK2	LIM domair	3985
ILMN_1785	1.52E-08	4.98E-11	-1.536229	TNFAIP6	TNF alpha	7130
ILMN_2364	1.55E-08	5.14E-11	-1.092336	SLC16A3	solute car	9123
ILMN_1728	1.62E-08	5.42E-11	-1.035041	IL17RA	interleuki	23765
ILMN_2391	1.65E-08	5.55E-11	-2.066555	FCGR1B	Fc fragmer	2210
ILMN_2367	1.71E-08	5.83E-11	-1.444445	CAMKK2	calcium/ca	10645
ILMN_1743	1.76E-08	6.10E-11	-1.329184	CAMKK2	calcium/ca	10645
ILMN_1861	1.76E-08	6.12E-11	-1.098548	PLXDC2	plexin dom	84898
ILMN_1769	1.76E-08	6.13E-11	-1.629113	PIWIL4	piwi like	143689
ILMN_1802	1.82E-08	6.49E-11	-1.068089	AGTRAP	angiotensi	57085
ILMN_1796	1.85E-08	6.68E-11	-1.787035	NLRC4	NLR family	58484
ILMN_1790	1.96E-08	7.09E-11	-1.320966	DDIAS	DNA damage	220042
ILMN_3251	2.04E-08	7.46E-11	-1.711959	LILRA6	leukocyte	79168
ILMN_2319	2.49E-08	9.36E-11	1.3474965	MATK	megakaryoc	4145
ILMN_1756	2.52E-08	9.55E-11	-1.219706	MUC1	mucin 1, c	4582
ILMN_1805	2.56E-08	9.75E-11	-1.921692	IFITM3	interferor	10410
ILMN_1779	2.89E-08	1.13E-10	-1.439401	HPSE	heparanase	10855
ILMN_1737	2.89E-08	1.14E-10	-1.511638	MAPK14	mitogen-ac	1432
ILMN_1693	3.01E-08	1.19E-10	-1.120588	VPS9D1	VPS9 domai	9605
ILMN_1774	3.25E-08	1.32E-10	-1.292053	IL1RN	interleuki	3557
ILMN_1748	3.29E-08	1.35E-10	-1.943075	S100A12	S100 calci	6283
ILMN_1750	3.50E-08	1.45E-10	-1.109219	S100A11	S100 calci	6282
ILMN_1659	3.58E-08	1.49E-10	-1.354369	LOC146880	Rho GTPase	146880
ILMN_2169	3.95E-08	1.70E-10	-2.312991	TDRD9	tudor doma	122402
ILMN_1811	4.21E-08	1.85E-10	-2.383898	TDRD9	tudor doma	122402
ILMN_2307	4.43E-08	1.97E-10	-1.004052	AGTRAP	angiotensi	57085
ILMN_3234	4.43E-08	1.97E-10	-1.019261	N4BP2L2	NEDD4 binc	10443
ILMN_3215	4.68E-08	2.10E-10	-1.707008	LINC00265	long inter	349114
ILMN_1690	4.76E-08	2.15E-10	-1.04513	SLC9A8	solute car	23315
ILMN_1731	4.97E-08	2.28E-10	-1.355335	CREB5	cAMP respc	9586
ILMN_2367	5.53E-08	2.63E-10	-1.178084	OSCAR	osteoclast	126014
ILMN_2124	5.53E-08	2.64E-10	-1.320975	SLC36A1	solute car	206358
ILMN_1707	5.53E-08	2.65E-10	-1.229768	NFIL3	nuclear fa	4783
ILMN_2062	6.16E-08	2.98E-10	1.6927768	PTGDR	prostaglar	5729
ILMN_1781	6.57E-08	3.27E-10	-1.006731	PPP1R3D	protein pł	5509
ILMN_1690	6.66E-08	3.33E-10	-1.161491	PDLIM7	PDZ and LI	9260
ILMN_1873	7.01E-08	3.52E-10	1.1477325	TNRC6C	trinucleot	57690
ILMN_1811	7.60E-08	3.85E-10	-1.671073	HOMER3	homer scaf	9454
ILMN_1740	9.11E-08	4.75E-10	-1.693514	SIGLEC5	sialic aci	8778
ILMN_1661	9.35E-08	4.96E-10	-1.569502	IRAK3	interleuki	11213
ILMN_1783	1.04E-07	5.58E-10	-1.050226	MIIP	migration	60672
ILMN_1902	1.05E-07	5.69E-10	-1.399544	LOC285147	uncharacte	285147

ILMN_1739	1.06E-07	5.75E-10	1.1199044	TNRC6A	trinucleot	27327
ILMN_1771	1.15E-07	6.27E-10	-1.139016	HAUS4	HAUS augmi	54930
ILMN_2202	1.16E-07	6.37E-10	-1.233154	FAR2	fatty acyl	55711
ILMN_1666	1.18E-07	6.50E-10	-1.127019	STK3	serine/thr	6788
ILMN_1808	1.20E-07	6.69E-10	-1.074859	SBNO2	strawberry	22904
ILMN_1766	1.22E-07	6.81E-10	-1.410012	BCAT1	branched c	586
ILMN_3243	1.24E-07	6.98E-10	-1.169617	DDX60L	DEAD-box h	91351
ILMN_1717	1.24E-07	7.03E-10	-1.170781	RNF24	ring finge	11237
ILMN_1711	1.25E-07	7.11E-10	-1.2462	ARHGAP26	Rho GTPase	23092
ILMN_1721	1.64E-07	9.74E-10	-1.528827	HIST1H3D	histone cl	8351
ILMN_1722	1.67E-07	9.94E-10	-2.584718	CASP5	caspase 5	838
ILMN_1663	1.67E-07	1.00E-09	-1.304441	RGL4	ral guanir	266747
ILMN_1748	1.73E-07	1.07E-09	-1.161133	CXCL14	C-X-C moti	9547
ILMN_1733	1.78E-07	1.11E-09	-1.003312	C1RL	complement	51279
ILMN_1709	1.87E-07	1.18E-09	-1.611178	F5	coagulatic	2153
ILMN_1763	1.90E-07	1.21E-09	2.3398288	CCR3	C-C motif	1232
ILMN_3245	1.98E-07	1.28E-09	1.0023624	SNORA61	small nucl	677838
ILMN_1724	1.98E-07	1.28E-09	-1.030392	GRN	granulin	2896
ILMN_2075	1.98E-07	1.28E-09	-1.33067	PGS1	phosphatic	9489
ILMN_1738	2.06E-07	1.36E-09	-2.139902	SLC1A3	solute car	6507
ILMN_1684	2.06E-07	1.37E-09	-1.00425	PXN	paxillin	5829
ILMN_1688	2.08E-07	1.38E-09	2.6079194	FCER1A	Fc fragmer	2205
ILMN_1726	2.09E-07	1.39E-09	-1.037821	CARD16	caspase re	114769
ILMN_1655	2.14E-07	1.43E-09	-1.390759	CDK5RAP2	CDK5 regul	55755
ILMN_2309	2.30E-07	1.58E-09	-1.344023	SMARCD3	SWI/SNF re	6604
ILMN_1714	2.53E-07	1.78E-09	-1.922748	MGAM	maltase-gl	8972
ILMN_1659	2.54E-07	1.80E-09	-1.03602	DNAJC3	DnaJ heat	5611
ILMN_1794	2.63E-07	1.89E-09	-1.145532	PGD	phosphoglu	5226
ILMN_1715	2.64E-07	1.90E-09	-1.082083	AQP9	aquaporin	366
ILMN_1790	3.25E-07	2.43E-09	-1.236944	CRISPLD2	cysteine r	83716
ILMN_3244	3.36E-07	2.53E-09	-1.175951	ASAP1-IT1	ASAP1 intr	29065
ILMN_1752	3.37E-07	2.57E-09	1.1277846	DHRS3	dehydroger	9249
ILMN_1765	3.37E-07	2.57E-09	-1.743429	ADGRG3	adhesion (	222487
ILMN_2409	3.37E-07	2.57E-09	-1.372606	CCPG1	cell cycle	9236
ILMN_2117	3.49E-07	2.72E-09	-1.228621	PLXNC1	plexin C1	10154
ILMN_1774	3.58E-07	2.81E-09	-4.477252	CD177	CD177 mole	57126
ILMN_1696	3.61E-07	2.84E-09	-1.311588	PYGL	phosphoryl	5836
ILMN_2186	3.62E-07	2.86E-09	-1.51095	PFKFB3	6-phosphof	5209
ILMN_2204	3.71E-07	2.98E-09	-1.374639	ST3GAL4	ST3 beta-g	6484
ILMN_1784	4.12E-07	3.37E-09	-1.006018	LILRB3	leukocyte	11025
ILMN_1689	4.21E-07	3.47E-09	-1.014528	C5AR1	complement	728
ILMN_1707	4.34E-07	3.60E-09	-1.267853	SORT1	sortilin 1	6272
ILMN_1685	4.34E-07	3.63E-09	-1.121893	ITGAM	integrin s	3684
ILMN_1732	4.65E-07	3.95E-09	-1.520165	SIPA1L2	signal inc	57568
ILMN_1693	4.71E-07	4.03E-09	-1.859966	CYP1B1	cytochrome	1545
ILMN_1750	4.78E-07	4.13E-09	-1.119496	MKNK1	MAP kinase	8569
ILMN_1699	4.97E-07	4.34E-09	1.9259245	TNFRSF21	TNF recept	27242

ILMN_1656З	5.08E-07	4.46E-09	-1.274343	GLT1D1	glycosyltı	144423
ILMN_1658З	5.21E-07	4.60E-09	1.2981605	KLRG1	killer cel	10219
ILMN_1651	5.23E-07	4.64E-09	-2.448379	COL17A1	collagen t	1308
ILMN_1668	5.38E-07	4.79E-09	-1.755679	SLC2A14	solute caı	144195
ILMN_1794	5.63E-07	5.05E-09	1.3225137	CTSW	cathepsin	1521
ILMN_1780	6.19E-07	5.65E-09	-1.863952	CLEC5A	C-type le	23601
ILMN_1762	6.35E-07	5.86E-09	-1.043427	C9orf72	chromosome	203228
ILMN_2044	8.09E-07	7.64E-09	1.6611351	NCR3	natural cy	259197
ILMN_2397	8.17E-07	7.74E-09	-1.088589	KDM6B	lysine dem	23135
ILMN_1776	8.67E-07	8.32E-09	-1.35164	RNASE4	ribonuclea	6038
ILMN_2321	8.91E-07	8.62E-09	1.3059317	P2RY10	purinergi	27334
ILMN_1684	9.50E-07	9.35E-09	-1.431438	ACSL1	acyl-CoA s	2180
ILMN_1794	9.73E-07	9.60E-09	-1.161327	GPAT3	glycerol-3	84803
ILMN_2055	1.00E-06	9.97E-09	1.6350089	KLRF1	killer cel	51348
ILMN_1651	1.00E-06	1.01E-08	-1.297736	HIST1H2BD	histone cl	3017
ILMN_2249	1.05E-06	1.07E-08	2.4357673	LRRC26	leucine ri	389816
ILMN_1687	1.04E-06	1.05E-08	-1.166121	VCAN	versican	1462
ILMN_1717	1.10E-06	1.13E-08	-1.078415	C9orf66	chromosome	157983
ILMN_2404	1.11E-06	1.14E-08	-1.030716	SERPINA1	serpin fan	5265
ILMN_1815	1.12E-06	1.15E-08	-1.709705	ADGRG3	adhesion (	222487
ILMN_1725	1.12E-06	1.16E-08	1.3705781	NELL2	neural EGI	4753
ILMN_2371	1.12E-06	1.17E-08	-1.605292	CEACAM1	carcinoemb	634
ILMN_1741	1.13E-06	1.17E-08	-1.283651	SLC11A1	solute caı	6556
ILMN_1777	1.25E-06	1.32E-08	-1.596114	C9orf106	chromosome	414318
ILMN_1776	1.32E-06	1.42E-08	1.243332	SCML1	sex comb c	6322
ILMN_1654	1.39E-06	1.51E-08	1.2314315	CD1C	CD1c molec	911
ILMN_1659	1.41E-06	1.53E-08	-1.179119	KIF1B	kinesin fa	23095
ILMN_2078	1.47E-06	1.61E-08	-1.104904	ALPK1	alpha kina	80216
ILMN_1767	1.56E-06	1.72E-08	-1.919973	CR1	complement	1378
ILMN_2169	1.56E-06	1.73E-08	-1.280798	TNNI2	troponin I	7136
ILMN_1788	1.62E-06	1.81E-08	-1.513477	MAPK14	mitogen-ac	1432
ILMN_1694	1.63E-06	1.83E-08	-1.35328	FAR2	fatty acyl	55711
ILMN_1671	1.68E-06	1.92E-08	-1.055832	CLIC4	chloride i	25932
ILMN_1675	1.69E-06	1.94E-08	-1.26688	PLPP2	phospholip	8612
ILMN_1664	1.76E-06	2.03E-08	-1.958991	CEACAM1	carcinoemb	634
ILMN_1742	1.81E-06	2.10E-08	-1.613293	CR1	complement	1378
ILMN_1789	1.81E-06	2.11E-08	1.2677185	TPM2	tropomyosi	7169
ILMN_1806	1.83E-06	2.14E-08	-1.484911	LIN7A	lin-7 homc	8825
ILMN_1913	1.83E-06	2.14E-08	-1.285438	IRAK3	interleuki	11213
ILMN_1796	1.84E-06	2.17E-08	-1.007937	NCF2	neutrophil	4688
ILMN_1709	1.89E-06	2.25E-08	1.1989946	EPHX2	epoxide hy	2053
ILMN_1669	1.92E-06	2.30E-08	1.9333478	MATK	megakaryoc	4145
ILMN_1691	1.95E-06	2.36E-08	-2.29932	G0S2	G0/G1 swit	50486
ILMN_2301	1.94E-06	2.33E-08	-1.259351	UBE2C	ubiquitin	11065
ILMN_1741	1.95E-06	2.35E-08	-1.065725	OSCAR	osteoclast	126014
ILMN_1656	1.96E-06	2.38E-08	-1.946294	NTN3	netrin 3	4917
ILMN_2066	2.00E-06	2.44E-08	-1.038625	TNFSF13B	tumor necı	10673

ILMN_1742(	2.05E-06	2.51E-08	1.9702749	CD160	CD160 mol€	11126
ILMN_17937	2.11E-06	2.61E-08	-1.033897	DIRC2	disrupted	84925
ILMN_16607	2.15E-06	2.67E-08	1.7981551	PPP2R2B	protein ph	5521
ILMN_17701	2.19E-06	2.74E-08	-1.046759	BST1	bone marrc	683
ILMN_21933	2.29E-06	2.90E-08	1.5010499	MMP23B	matrix met	8510
ILMN_16608	2.34E-06	2.98E-08	-1.352142	PFKFB3	6-phosphof	5209
ILMN_21147	2.38E-06	3.07E-08	-2.288611	SLPI	secretory	6590
ILMN_23881	2.51E-06	3.29E-08	-1.564626	CR1	complement	1378
ILMN_20535	2.51E-06	3.30E-08	-1.078587	PARP9	poly(ADP-r	83666
ILMN_23589	2.54E-06	3.34E-08	-1.299367	TP53I3	tumor prot	9540
ILMN_17998	2.59E-06	3.43E-08	-2.194926	ANKRD22	ankyrin re	118932
ILMN_18149	2.59E-06	3.45E-08	-1.045068	PDLIM7	PDZ and LI	9260
ILMN_16922	2.65E-06	3.54E-08	-1.095043	DOK3	docking pr	79930
ILMN_21745	2.73E-06	3.68E-08	-1.423979	HNRNPA3P1	heterogene	10151
ILMN_17457	2.75E-06	3.71E-08	1.2571424	POLL	polymerase	27343
ILMN_16602	2.77E-06	3.76E-08	-1.227666	LINC00999	long inter	399744
ILMN_16514	2.87E-06	3.92E-08	-1.657507	GADD45G	growth arr	10912
ILMN_17081	3.08E-06	4.25E-08	-1.367464	TMEM144	transmembr	55314
ILMN_17239	3.14E-06	4.35E-08	1.3378551	TARP	TCR gamma	445347
ILMN_17459	3.17E-06	4.40E-08	-1.189392	GAS7	growth arr	8522
ILMN_17162	3.29E-06	4.64E-08	1.2104501	CCL4L1	C-C motif	388372
ILMN_17989	3.31E-06	4.69E-08	-1.725889	ADGRG3	adhesion (	222487
ILMN_17501	3.43E-06	4.87E-08	1.2228505	TESC	tescalcin	54997
ILMN_32305	3.47E-06	4.97E-08	-1.306954	LINC00152	long inter	112597
ILMN_17803	3.46E-06	4.95E-08	-1.04927	KCNJ2	potassium	3759
ILMN_21594	3.48E-06	5.00E-08	-1.059952	STXBP2	syntaxin b	6813
ILMN_17871	3.57E-06	5.15E-08	2.5650231	NOV	nephroblas	4856
ILMN_23932	3.53E-06	5.08E-08	-1.226047	GK	glycerol k	2710
ILMN_16905	3.57E-06	5.17E-08	1.2244809	GZMM	granzyme M	3004
ILMN_16848	3.60E-06	5.21E-08	-1.267609	SAMSN1	SAM domair	64092
ILMN_18064	3.70E-06	5.42E-08	-1.822419	NTNG2	netrin G2	84628
ILMN_17487	3.68E-06	5.35E-08	-1.295805	CTRC	chymotryps	11330
ILMN_1702(	3.79E-06	5.58E-08	-1.205235	SV2A	synaptic v	9900
ILMN_23867	3.84E-06	5.70E-08	1.7431212	KLRC3	killer cel	3823
ILMN_18131	3.86E-06	5.74E-08	-1.033623	TOM1	target of	10043
ILMN_1730(	3.91E-06	5.82E-08	-1.413342	RNASE2	ribonuclea	6036
ILMN_18089	3.91E-06	5.84E-08	-1.492322	CLEC4D	C-type lec	338339
ILMN_19101	3.91E-06	5.85E-08	1.4780056	RRN3P1	RRN3 homol	730092
ILMN_16855	4.18E-06	6.29E-08	1.6318895	KLRF1	killer cel	51348
ILMN_16757	4.21E-06	6.34E-08	-1.216808	KCNJ15	potassium	3772
ILMN_17395	4.49E-06	6.83E-08	-1.422529	DPRXP4	divergent-	503645
ILMN_16951	4.52E-06	6.88E-08	-1.657312	CA4	carbonic a	762
ILMN_17025	4.74E-06	7.22E-08	1.1536452	CD244	CD244 mole	51744
ILMN_17605	4.78E-06	7.32E-08	1.2710121	EOMES	eomesoderm	8320
ILMN_17065	4.83E-06	7.41E-08	-1.057846	EIF2AK2	eukaryotic	5610
ILMN_16559	4.99E-06	7.72E-08	-1.326996	ELL2	elongatior	22936
ILMN_17991	5.24E-06	8.15E-08	1.4719649	KLRD1	killer cel	3824

ILMN_2256(	5.29E-06	8.29E-08	-1.820729	SERPINA1	serpin fam	5265
ILMN_1671{	5.48E-06	8.62E-08	1.2377955	SEPT1	septin 1	1731
ILMN_1758(	5.77E-06	9.26E-08	-1.237414	HIST1H2BD	histone cl	3017
ILMN_1862(	5.98E-06	9.59E-08	1.1539447	RORA	RAR relate	6095
ILMN_1797(	6.05E-06	9.76E-08	1.5698221	KLRD1	killer cel	3824
ILMN_2316	6.13E-06	9.95E-08	1.2154872	HOPX	HOP homeob	84525
ILMN_2244(	6.32E-06	1.03E-07	1.1404123	LBH	limb bud a	81606
ILMN_1749(	6.35E-06	1.04E-07	-1.044766	TIMP2	TIMP metal	7077
ILMN_1732	6.41E-06	1.05E-07	1.2841974	ID3	inhibitor	3399
ILMN_1733	6.52E-06	1.07E-07	-1.123925	LOXL3	lysyl oxic	84695
ILMN_1725	6.58E-06	1.08E-07	-1.244976	GK	glycerol k	2710
ILMN_1684	6.65E-06	1.10E-07	1.179611	IL2RB	interleuki	3560
ILMN_1812(	7.80E-06	1.33E-07	1.2834805	ABCB1	ATP bindir	5243
ILMN_2379(	7.80E-06	1.33E-07	-1.409989	FCAR	Fc fragmer	2204
ILMN_1686	7.83E-06	1.34E-07	-1.091181	TIFA	TRAF inter	92610
ILMN_2073	7.97E-06	1.37E-07	1.8242404	S1PR5	sphingosir	53637
ILMN_2092	8.07E-06	1.38E-07	-1.66066	GPR141	G protein-	353345
ILMN_2394	8.12E-06	1.40E-07	1.0256397	PLEKHA1	pleckstrir	59338
ILMN_2062(	8.30E-06	1.44E-07	-1.134148	HOOK3	hook micrc	84376
ILMN_1712	8.40E-06	1.46E-07	-2.740079	CEACAM6	carcinoemb	4680
ILMN_1792(	8.40E-06	1.46E-07	-1.471063	MCTP2	multiple (	55784
ILMN_1701	8.48E-06	1.48E-07	1.4980664	SH2D1B	SH2 domair	117157
ILMN_2370	8.65E-06	1.51E-07	-1.036928	SLC24A4	solute car	123041
ILMN_1699	8.65E-06	1.51E-07	-1.284588	ST14	suppressic	6768
ILMN_1808	8.94E-06	1.57E-07	-2.081349	PNPLA1	patatin li	285848
ILMN_2142	9.17E-06	1.62E-07	-1.526277	MANSC1	MANSC doma	54682
ILMN_1764	9.23E-06	1.63E-07	1.0338735	OXNAD1	oxidoreduc	92106
ILMN_1716	9.42E-06	1.68E-07	-1.435839	CEACAM1	carcinoemb	634
ILMN_2408(	9.49E-06	1.69E-07	-1.461652	SMARCD3	SWI/SNF re	6604
ILMN_1704	9.51E-06	1.70E-07	-1.601658	PGLYRP1	peptidogly	8993
ILMN_1796	9.73E-06	1.75E-07	-1.778055	BMX	BMX non-re	660
ILMN_1711	1.08E-05	1.96E-07	1.1500978	CD96	CD96 molec	10225
ILMN_1770(	1.12E-05	2.05E-07	1.3613034	KLHL3	kelch like	26249
ILMN_1691	1.14E-05	2.10E-07	-1.369782	JAG1	jagged 1	182
ILMN_1731(	1.16E-05	2.17E-07	1.2666751	ABLIM1	actin binc	3983
ILMN_1696	1.19E-05	2.22E-07	-1.142875	ROPN1L	rhophilin	83853
ILMN_2199	1.20E-05	2.25E-07	-1.091411	SLITRK4	SLIT and N	139065
ILMN_1796	1.22E-05	2.30E-07	1.4473529	CLIC3	chloride i	9022
ILMN_1701(	1.22E-05	2.31E-07	1.6046577	DCANP1	dendritic	140947
ILMN_1657{	1.25E-05	2.37E-07	-1.024648	TLR8	toll like	51311
ILMN_1734	1.26E-05	2.39E-07	1.1061089	TC2N	tandem C2	123036
ILMN_1761	1.28E-05	2.44E-07	-1.469624	CYSTM1	cysteine r	84418
ILMN_2352(	1.29E-05	2.48E-07	1.4945866	ADGRG1	adhesion (	9289
ILMN_1726	1.33E-05	2.57E-07	1.1227319	SLC45A3	solute car	85414
ILMN_1659(	1.36E-05	2.64E-07	-1.488634	RSPH9	radial spc	221421
ILMN_1717	1.38E-05	2.70E-07	-1.124395	MMP25	matrix met	64386
ILMN_1699	1.38E-05	2.71E-07	1.1768064	PPIAL4C	peptidylpr	653598

ILMN_17952	1.47E-05	2.92E-07	-1.087155	SIGLEC9	sialic aci	27180
ILMN_22898	1.47E-05	2.93E-07	-1.030659	FCGR2A	Fc fragmer	2212
ILMN_16880	1.54E-05	3.08E-07	1.0395837	TBC1D4	TBC1 domai	9882
ILMN_21932	1.57E-05	3.14E-07	-1.974659	DEFA1	defensin a	1667
ILMN_23430	1.57E-05	3.15E-07	1.3047991	NCALD	neurocalci	83988
ILMN_17991	1.60E-05	3.22E-07	-1.173083	MARC1	mitochondr	64757
ILMN_17619	1.64E-05	3.33E-07	1.828765	FGFBP2	fibroblast	83888
ILMN_17694	1.64E-05	3.33E-07	1.0245249	TMEM261	transmembr	90871
ILMN_21682	1.67E-05	3.39E-07	1.1798663	GPR183	G protein-	1880
ILMN_23841	1.81E-05	3.76E-07	1.4951728	ADGRG1	adhesion (	9289
ILMN_23297	1.86E-05	3.91E-07	-1.135248	ECM1	extracellu	1893
ILMN_16524	1.92E-05	4.04E-07	-1.39831	MANSC1	MANSC doma	54682
ILMN_17129	1.92E-05	4.05E-07	-1.231933	NQO2	NAD(P)H qu	4835
ILMN_16559	2.07E-05	4.46E-07	-1.273689	STAB1	stabilin 1	23166
ILMN_17144	2.07E-05	4.47E-07	1.208528	KLF12	Kruppel li	11278
ILMN_17452	2.11E-05	4.57E-07	-1.435474	PLSCR1	phospholip	5359
ILMN_32410	2.16E-05	4.70E-07	1.7466602	MYBL1	MYB proto-	4603
ILMN_17805	2.19E-05	4.79E-07	-1.339561	OSM	oncostatin	5008
ILMN_16798	2.21E-05	4.82E-07	-1.279162	CST7	cystatin F	8530
ILMN_17130	2.23E-05	4.89E-07	-1.036157	PSTPIP2	proline-se	9050
ILMN_17584	2.24E-05	4.90E-07	-1.035089	TNFSF13B	tumor necr	10673
ILMN_18595	2.36E-05	5.22E-07	-1.528311	LOC1019301	uncharacte	101930164
ILMN_17817	2.38E-05	5.30E-07	-1.689553	IL18R1	interleuki	8809
ILMN_17413	2.41E-05	5.38E-07	-1.040774	B3GNT8	UDP-GlcNAc	374907
ILMN_17261	2.41E-05	5.39E-07	-1.198092	ITGAX	integrin s	3687
ILMN_17638	2.47E-05	5.52E-07	-1.168234	ANPEP	alanyl ami	290
ILMN_16885	2.49E-05	5.59E-07	-1.567133	CAMP	cathelicic	820
ILMN_20522	2.50E-05	5.62E-07	-1.240269	GADD45A	growth arr	1647
ILMN_16621	2.60E-05	5.89E-07	1.0131734	ZNF248	zinc finge	57209
ILMN_17667	2.65E-05	6.04E-07	-2.312137	BPI	bactericic	671
ILMN_16696	2.78E-05	6.38E-07	-1.424898	GRB10	growth fac	2887
ILMN_17080	2.81E-05	6.49E-07	1.1314076	PLEKHF1	pleckstrin	79156
ILMN_16568	2.97E-05	6.92E-07	-1.776762	GPR141	G protein-	353345
ILMN_17231	3.01E-05	7.06E-07	-1.12632	CDKN2B	cyclin dep	1030
ILMN_22996	3.04E-05	7.13E-07	1.225798	TNFRSF25	TNF recept	8718
ILMN_17974	3.07E-05	7.22E-07	1.5652933	FCRL3	Fc recepto	115352
ILMN_17159	3.21E-05	7.63E-07	1.6861086	FBXO7	F-box prot	25793
ILMN_17201	3.28E-05	7.81E-07	-1.039055	ETS2	ETS proto-	2114
ILMN_18052	3.32E-05	7.93E-07	-1.225745	LRG1	leucine ri	116844
ILMN_16538	3.33E-05	7.98E-07	-1.066531	NAMPT	nicotinami	10135
ILMN_17912	3.35E-05	8.03E-07	-1.23513	FBXL13	F-box and	222235
ILMN_16782	3.35E-05	8.04E-07	1.4408343	ZNF683	zinc finge	257101
ILMN_18002	3.44E-05	8.34E-07	-1.771479	PPARG	peroxisome	5468
ILMN_16913	3.39E-05	8.17E-07	-1.131873	NME8	NME/NM23 f	51314
ILMN_17122	3.52E-05	8.60E-07	-1.408995	PPP1R3B	protein ph	79660
ILMN_20948	3.62E-05	8.90E-07	1.3213398	ABCB1	ATP bindir	5243
ILMN_18131	3.57E-05	8.74E-07	-1.194076	ANKDD1A	ankyrin re	348094

ILMN_1722[illegible]	3.80E-05	9.43E-07	-2.130677	GALNT14	polypeptic	79623
ILMN_1745[illegible]	3.71E-05	9.13E-07	1.198207	FBXO7	F-box prot	25793
ILMN_2342[illegible]	3.75E-05	9.27E-07	-1.323783	ASGR2	asialoglyc	433
ILMN_1735[illegible]	4.00E-05	1.01E-06	-2.598524	SLC11A1	solute car	6556
ILMN_3251[illegible]	4.08E-05	1.03E-06	-4.751646	CD177	CD177 mole	57126
ILMN_2256[illegible]	3.88E-05	9.66E-07	1.6648718	GNLY	granulysin	10578
ILMN_1754[illegible]	3.90E-05	9.75E-07	1.7352134	CACNA2D3	calcium vc	55799
ILMN_1667[illegible]	3.91E-05	9.76E-07	-1.045732	FAM129A	family wit	116496
ILMN_1725[illegible]	3.95E-05	9.90E-07	-2.207416	DEFA1B	defensin a	728358
ILMN_2227[illegible]	4.00E-05	1.01E-06	1.5139364	CCDC65	coiled-coi	85478
ILMN_1797[illegible]	4.01E-05	1.01E-06	-1.033061	FFAR2	free fatty	2867
ILMN_1692[illegible]	4.04E-05	1.02E-06	1.033653	GNA12	G protein	2768
ILMN_1797[illegible]	4.21E-05	1.08E-06	1.8551895	PRSS23	protease,	11098
ILMN_1716[illegible]	4.40E-05	1.13E-06	-1.49761	FOXM1	forkhead b	2305
ILMN_2358[illegible]	4.24E-05	1.08E-06	-1.162357	NBN	nibrin	4683
ILMN_2360[illegible]	4.61E-05	1.20E-06	-1.240929	CAV2	caveolin 2	858
ILMN_1789[illegible]	4.92E-05	1.30E-06	-3.35364	SLC51A	solute car	200931
ILMN_1775[illegible]	4.66E-05	1.22E-06	-1.152043	PROK2	prokinetic	60675
ILMN_1694[illegible]	4.67E-05	1.22E-06	-1.282546	ASGR2	asialoglyc	433
ILMN_2083[illegible]	4.72E-05	1.24E-06	-1.257622	TGFA	transformi	7039
ILMN_1732[illegible]	4.75E-05	1.25E-06	1.2374089	DPM2	dolichyl-p	8818
ILMN_1757[illegible]	5.06E-05	1.34E-06	1.318875	TPM2	tropomyosi	7169
ILMN_1678[illegible]	5.12E-05	1.37E-06	-1.535517	UPB1	beta-ureic	51733
ILMN_1781[illegible]	5.17E-05	1.39E-06	-1.306235	TUFT1	tuftelin 1	7286
ILMN_2355[illegible]	5.30E-05	1.43E-06	-1.056886	MGST1	microsomal	4257
ILMN_1772[illegible]	5.33E-05	1.44E-06	-1.188029	TLR2	toll like	7097
ILMN_2205[illegible]	5.37E-05	1.46E-06	-1.556676	NSUN7	NOP2/Sun F	79730
ILMN_1676[illegible]	5.43E-05	1.48E-06	-1.151065	DISC1	disrupted	27185
ILMN_1656[illegible]	5.67E-05	1.56E-06	-2.12278	TRIM6	tripartite	117854
ILMN_2327[illegible]	5.43E-05	1.48E-06	1.8984121	IL5RA	interleuki	3568
ILMN_3237[illegible]	5.56E-05	1.52E-06	1.1126533	CARNS1	carnosine	57571
ILMN_1766[illegible]	5.65E-05	1.55E-06	1.3383646	CPA3	carboxypep	1359
ILMN_3241[illegible]	5.76E-05	1.59E-06	1.0934059	TMEM204	transmembr	79652
ILMN_2132[illegible]	5.76E-05	1.60E-06	-1.549638	ANKRD22	ankyrin re	118932
ILMN_1719[illegible]	5.82E-05	1.61E-06	1.0437378	ZAP70	zeta chain	7535
ILMN_1788[illegible]	5.84E-05	1.62E-06	1.2340758	NCALD	neurocalci	83988
ILMN_2302[illegible]	5.84E-05	1.62E-06	1.3472263	FCGBP	Fc fragmer	8857
ILMN_1798[illegible]	6.19E-05	1.74E-06	1.1409951	GPR183	G protein-	1880
ILMN_1806[illegible]	6.25E-05	1.76E-06	1.0795553	CSTF3	cleavage s	1479
ILMN_2165[illegible]	6.34E-05	1.79E-06	-2.147023	DEFA3	defensin a	1668
ILMN_2409[illegible]	6.50E-05	1.85E-06	1.0272151	PRR5	proline ri	55615
ILMN_2267[illegible]	6.53E-05	1.86E-06	1.0983917	HPS1	HPS1, biog	3257
ILMN_1676[illegible]	6.76E-05	1.93E-06	-1.06838	ADCY3	adenylate	109
ILMN_1689[illegible]	6.97E-05	2.01E-06	-1.106298	RBM47	RNA bindir	54502
ILMN_1712[illegible]	7.15E-05	2.07E-06	-1.292944	FAM20C	family wit	56975
ILMN_1728[illegible]	7.24E-05	2.11E-06	-1.034189	CXCL16	C-X-C moti	58191
ILMN_1775[illegible]	7.26E-05	2.12E-06	1.4001071	ARL4A	ADP ribosy	10124

ILMN_17571	7.33E-05	2.15E-06	-1.335208	TMEM88	transmembr	92162
ILMN_17803	7.60E-05	2.25E-06	1.1647913	GPR18	G protein-	2841
ILMN_16897	7.92E-05	2.36E-06	-1.054078	IL1RN	interleuki	3557
ILMN_18032	7.98E-05	2.38E-06	-1.158421	TMC4	transmembr	147798
ILMN_21027	8.10E-05	2.43E-06	-2.372872	DEFA1B	defensin a	728358
ILMN_19122	8.32E-05	2.51E-06	-1.084343	SUCNR1	succinate	56670
ILMN_17790	8.54E-05	2.58E-06	1.7236175	CACNG6	calcium vo	59285
ILMN_16988	8.50E-05	2.57E-06	-1.431902	ZAK	sterile al	51776
ILMN_17983	8.66E-05	2.63E-06	1.0969359	ACKR3	atypical c	57007
ILMN_16723	8.68E-05	2.64E-06	-1.379443	BMX	BMX non-re	660
ILMN_31815	9.05E-05	2.78E-06	-1.102045	C10orf105	chromosome	414152
ILMN_17192	9.26E-05	2.88E-06	-1.226795	DISC1	disrupted	27185
ILMN_23829	9.15E-05	2.83E-06	1.2388419	CCDC7	coiled-coi	79741
ILMN_16575	9.21E-05	2.86E-06	1.1057727	TSEN54	tRNA splic	283989
ILMN_16523	9.33E-05	2.91E-06	-1.036823	SIGLEC5	sialic aci	8778
ILMN_17156	9.66E-05	3.03E-06	1.3363439	IL23A	interleuki	51561
ILMN_16793	9.89E-05	3.12E-06	-2.233789	DEFA1B	defensin a	728358
ILMN_23356	1.00E-04	3.17E-06	1.0656041	SKAP1	src kinase	8631
ILMN_20485	1.00E-04	3.19E-06	1.5163938	LRRN3	leucine ri	54674
ILMN_17411	1.01E-04	3.22E-06	1.0406687	TXK	TXK tyrosi	7294
ILMN_22292	1.10E-04	3.57E-06	-1.53947	SIGLEC16	sialic aci	400709
ILMN_33078	1.10E-04	3.58E-06	1.7751288	CHI3L1	chitinase	1116
ILMN_24135	1.13E-04	3.68E-06	1.8268064	HEMGN	hemogen	55363
ILMN_17526	1.16E-04	3.82E-06	1.1394716	KLRC1	killer cel	3821
ILMN_16751	1.17E-04	3.84E-06	-1.828427	RETN	resistin	56729
ILMN_17051	1.18E-04	3.88E-06	-2.285955	MPO	myeloperox	4353
ILMN_16669	1.18E-04	3.91E-06	1.2196489	ADGRG5	adhesion G	221188
ILMN_17726	1.26E-04	4.21E-06	1.4322057	NSG1	neuron spe	27065
ILMN_17781	1.26E-04	4.23E-06	-1.369093	ZMYND15	zinc finge	84225
ILMN_17603	1.26E-04	4.24E-06	1.1568707	CD8A	CD8a molec	925
ILMN_17649	1.29E-04	4.34E-06	-1.985165	SERPINA1	serpin fam	5265
ILMN_20747	1.33E-04	4.50E-06	1.6840558	FCRL6	Fc recepto	343413
ILMN_18048	1.31E-04	4.40E-06	-1.168705	LSMEM1	leucine ri	286006
ILMN_16574	1.34E-04	4.53E-06	-1.15692	CEACAM4	carcinoemb	1089
ILMN_16958	1.38E-04	4.71E-06	1.3524558	KRT72	keratin 72	140807
ILMN_32431	1.39E-04	4.75E-06	1.65413	ADGRE4P	adhesion G	326342
ILMN_17516	1.39E-04	4.75E-06	1.3526141	FOSB	FosB proto	2354
ILMN_16792	1.39E-04	4.78E-06	1.8363721	TGM2	transgluta	7052
ILMN_17018	1.39E-04	4.78E-06	-1.157417	KLHL2	kelch like	11275
ILMN_17217	1.43E-04	4.93E-06	-1.235283	IL18RAP	interleuki	8807
ILMN_16974	1.47E-04	5.07E-06	1.0917579	PRR5L	proline ri	79899
ILMN_16614	1.55E-04	5.39E-06	-1.065677	ATP11C	ATPase pho	286410
ILMN_17500	1.55E-04	5.43E-06	1.1744806	CD200R1	CD200 rece	131450
ILMN_23969	1.56E-04	5.47E-06	-1.165487	KCNJ15	potassium	3772
ILMN_24126	1.57E-04	5.53E-06	-1.058038	TP53I11	tumor prot	9537
ILMN_16990	1.60E-04	5.65E-06	1.0459183	MAP3K7CL	MAP3K7 C-t	56911
ILMN_17819	1.60E-04	5.67E-06	-1.346471	MGST1	microsomal	4257

ILMN_2343(	1.60E-04	5.67E-06	1.2630269	SAMD3	sterile al	154075
ILMN_23841	1.70E-04	6.11E-06	1.6148399	KLRC1	killer cel	3821
ILMN_1773(	1.68E-04	6.00E-06	1.3254562	LRRN3	leucine ri	54674
ILMN_17107	1.71E-04	6.16E-06	1.1478828	GZMK	granzyme K	3003
ILMN_17643	1.73E-04	6.24E-06	-1.173217	HDAC4	histone de	9759
ILMN_17818	1.79E-04	6.49E-06	1.7266887	FASLG	Fas ligand	356
ILMN_1660(	1.80E-04	6.57E-06	1.0115056	PRR5	proline ri	55615
ILMN_17679	1.83E-04	6.67E-06	-1.455791	NSUN7	NOP2/Sun F	79730
ILMN_23031	1.89E-04	6.94E-06	1.2795194	MBNL3	muscleblir	55796
ILMN_18122	2.05E-04	7.63E-06	1.1666471	LY9	lymphocyte	4063
ILMN_17511	2.18E-04	8.28E-06	-1.371205	COL7A1	collagen t	1294
ILMN_22521	2.20E-04	8.39E-06	-1.439208	UBC	ubiquitin	7316
ILMN_17382	2.10E-04	7.88E-06	-1.254152	TREML3P	triggering	340206
ILMN_17312	2.11E-04	7.92E-06	1.3518956	GZMH	granzyme H	2999
ILMN_1753(	2.14E-04	8.08E-06	1.0507971	TRIM51	tripartite	84767
ILMN_16853	2.16E-04	8.18E-06	-1.095845	PSG3	pregnancy	5671
ILMN_17062	2.19E-04	8.32E-06	1.6308005	PTGDR	prostaglar	5729
ILMN_23224	2.22E-04	8.44E-06	1.063121	RORA	RAR relate	6095
ILMN_16779	2.25E-04	8.57E-06	-2.249104	LTF	lactotrans	4057
ILMN_17923	2.30E-04	8.80E-06	1.6919032	HDC	histidine	3067
ILMN_1749(	2.36E-04	9.09E-06	1.0839637	HLA-DPB1	major hist	3115
ILMN_23795	2.42E-04	9.36E-06	-1.345765	CD163	CD163 mole	9332
ILMN_16631	2.46E-04	9.49E-06	-1.101964	DSC2	desmocolli	1824
ILMN_32502	2.56E-04	9.98E-06	-1.416523	ACVRL1	activin A	94
ILMN_17712	2.54E-04	9.85E-06	-1.245094	SKA1	spindle ar	220134
ILMN_1694(	2.56E-04	9.96E-06	-1.039973	GADD45A	growth arr	1647
ILMN_17661	2.58E-04	1.00E-05	1.3111699	FHIT	fragile hi	2272
ILMN_23732	2.95E-04	1.19E-05	-2.460406	MSR1	macrophage	4481
ILMN_16548	2.67E-04	1.05E-05	1.7044445	CLC	Charcot-Le	1178
ILMN_2384(	2.80E-04	1.12E-05	-2.048534	GPER1	G protein-	2852
ILMN_17449	2.72E-04	1.08E-05	1.6067652	RHOBTB3	Rho relate	22836
ILMN_16995	2.77E-04	1.10E-05	1.2678266	FCRL3	Fc recepto	115352
ILMN_17183	2.83E-04	1.13E-05	-1.025533	NECTIN2	nectin cel	5819
ILMN_23383	2.88E-04	1.15E-05	1.0817164	UBASH3A	ubiquitin	53347
ILMN_1722(	2.94E-04	1.18E-05	-1.275876	CD163	CD163 mole	9332
ILMN_21431	3.05E-04	1.24E-05	-1.819867	KIF11	kinesin fa	3832
ILMN_17395	3.27E-04	1.35E-05	-1.425224	CYB5R2	cytochrome	51700
ILMN_17964	3.27E-04	1.35E-05	-2.297493	C1QB	complement	713
ILMN_1793(	3.11E-04	1.26E-05	1.0178317	SERPINA10	serpin fam	51156
ILMN_1658(	3.16E-04	1.29E-05	1.1269222	DTX3	deltex E3	196403
ILMN_17204	3.58E-04	1.52E-05	-2.637548	LONRF3	LON peptic	79836
ILMN_23841	3.23E-04	1.32E-05	1.7127725	KLRC1	killer cel	3821
ILMN_1728(	3.23E-04	1.33E-05	-1.073482	CREB5	cAMP respo	9586
ILMN_32478	3.28E-04	1.35E-05	1.1967786	PCED1B-AS1	PCED1B ant	100233209
ILMN_1680(	3.28E-04	1.36E-05	1.9947866	HEMGN	hemogen	55363
ILMN_2400(	3.31E-04	1.37E-05	1.0156998	ZNF626	zinc finge	199777
ILMN_1781(	3.64E-04	1.55E-05	-2.148223	SYN2	synapsin I	6854

ILMN_1731(	3.38E-04	1.41E-05	1.0875488	LY9	lymphocyte	4063
ILMN_1695(	3.53E-04	1.49E-05	-1.62339	KIF20A	kinesin fa	10112
ILMN_1706(	3.64E-04	1.55E-05	-1.690801	RFX2	regulatory	5990
ILMN_1658(	3.92E-04	1.72E-05	-1.766663	CENPI	centromere	2491
ILMN_16721	3.61E-04	1.54E-05	1.2981097	KRT73	keratin 73	319101
ILMN_1779(	3.64E-04	1.56E-05	1.0201936	AK1	adenylate	203
ILMN_1799(	3.69E-04	1.58E-05	1.3717623	NOXA1	NADPH oxic	10811
ILMN_1751(	3.72E-04	1.60E-05	1.013135	TMEM204	transmembı	79652
ILMN_1690(	3.83E-04	1.66E-05	1.7248404	CNTNAP2	contactin	26047
ILMN_1751(	4.24E-04	1.89E-05	-2.557843	NEDD4	neural pre	4734
ILMN_1740(	3.77E-04	1.63E-05	1.0751071	PRF1	perforin 1	5551
ILMN_1664(	3.88E-04	1.69E-05	1.6135001	PTGDS	prostaglar	5730
ILMN_1672(	3.88E-04	1.69E-05	1.0645736	EPHA4	EPH recept	2043
ILMN_1706(	3.91E-04	1.71E-05	-2.255674	ELANE	elastase,	1991
ILMN_2064(	4.32E-04	1.93E-05	-2.359015	METTL7B	methyltrar	196410
ILMN_2291(	3.99E-04	1.76E-05	1.3372082	FAM102A	family wit	399665
ILMN_1770(	4.03E-04	1.78E-05	1.0440039	BEX3	brain expı	27018
ILMN_1701(	4.07E-04	1.80E-05	-1.021654	SCO2	SCO2 cytoc	9997
ILMN_1703(	4.24E-04	1.89E-05	1.8444184	PTGDR2	prostaglar	11251
ILMN_1808(	4.47E-04	2.01E-05	-1.089354	KIF14	kinesin fa	9928
ILMN_2125(	4.70E-04	2.13E-05	1.2297429	TIGIT	T-cell imn	201633
ILMN_2383(	4.78E-04	2.18E-05	1.1292325	ZNF74	zinc finge	7625
ILMN_2116(	5.53E-04	2.58E-05	-3.482899	OLFM4	olfactomec	10562
ILMN_1679(	5.07E-04	2.33E-05	-1.031453	EXOC6	exocyst cc	54536
ILMN_1708(	5.02E-04	2.30E-05	1.6327785	PDZK1IP1	PDZK1 inte	10158
ILMN_17861	5.07E-04	2.33E-05	-1.102847	CCNA2	cyclin A2	890
ILMN_1658(	5.10E-04	2.35E-05	1.1015155	ZNF831	zinc finge	128611
ILMN_2258(	5.12E-04	2.36E-05	1.0269393	SAMD3	sterile al	154075
ILMN_1727(	5.44E-04	2.53E-05	1.9534122	OLIG2	oligodendı	10215
ILMN_2234(	6.09E-04	2.89E-05	-3.039436	BEX1	brain expı	55859
ILMN_1699(	5.39E-04	2.50E-05	-1.007646	ZNF516	zinc finge	9658
ILMN_1656(	5.53E-04	2.58E-05	-1.223031	PLAU	plasminoge	5328
ILMN_2379(	5.44E-04	2.53E-05	1.1729348	HAGH	hydroxyacy	3029
ILMN_1776(	5.54E-04	2.59E-05	1.3000492	PARP15	poly(ADP-ı	165631
ILMN_1748(	5.70E-04	2.67E-05	1.3184034	CD8B	CD8b molec	926
ILMN_1711(	6.44E-04	3.10E-05	-2.156331	CDC25A	cell divis	993
ILMN_1756(	5.90E-04	2.78E-05	1.1540521	RTN1	reticulon	6252
ILMN_1758(	6.38E-04	3.07E-05	-1.692606	QSOX1	quiescin s	5768
ILMN_23521	6.06E-04	2.87E-05	1.2510139	ERBB2	erb-b2 rec	2064
ILMN_1757(	6.83E-04	3.34E-05	-1.865691	NEIL3	nei like I	55247
ILMN_1742(	6.30E-04	3.02E-05	1.0168426	PYHIN1	pyrin and	149628
ILMN_23541	6.37E-04	3.06E-05	1.2076902	CD8B	CD8b molec	926
ILMN_1668(	6.66E-04	3.24E-05	1.3392277	SPNS3	sphingolip	201305
ILMN_2295(	6.73E-04	3.28E-05	1.0208763	NGDN	neuroguidi	25983
ILMN_1725(	6.79E-04	3.32E-05	1.0618556	SLC27A5	solute car	10998
ILMN_1670(	6.99E-04	3.44E-05	1.0588971	OPN3	opsin 3	23596
ILMN_20491	7.13E-04	3.52E-05	1.5572954	DNASE1L3	deoxyribor	1776

ILMN_17052	7.19E-04	3.56E-05	-1.173462	TMEM110	transmembr	375346
ILMN_16914	7.38E-04	3.67E-05	-1.355628	BAMBI	BMP and ac	25805
ILMN_17990	7.39E-04	3.68E-05	1.3419264	TSPAN5	tetraspani	10098
ILMN_17405	7.75E-04	3.90E-05	1.0063971	TMSB15B	thymosin b	286527
ILMN_18060	7.97E-04	4.04E-05	-2.490911	CEACAM8	carcinoemb	1088
ILMN_17040	9.17E-04	4.77E-05	-2.057263	OGFOD2	2-oxogluta	79676
ILMN_17963	7.94E-04	4.01E-05	-1.049832	SMPDL3A	sphingomye	10924
ILMN_16818	8.17E-04	4.15E-05	1.1634915	ZNF540	zinc finge	163255
ILMN_17991	9.67E-04	5.08E-05	-3.444353	COL17A1	collagen t	1308
ILMN_21639	7.72E-04	3.88E-05	-4.440877	OXGR1	oxoglutara	27199
ILMN_17008	8.58E-04	4.40E-05	-1.46813	SLC27A2	solute car	11001
ILMN_17011	8.30E-04	4.23E-05	1.5474541	PLA2G7	phospholip	7941
ILMN_17451	8.30E-04	4.23E-05	1.0123812	FAM102A	family wit	399665
ILMN_22514	9.17E-04	4.77E-05	-1.872238	RPS6KA1	ribosomal	6195
ILMN_17979	8.42E-04	4.30E-05	1.140012	EXTL2	exostosin	2135
ILMN_17160	8.85E-04	4.57E-05	1.6526711	MYL4	myosin lig	4635
ILMN_32372	1.04E-03	5.54E-05	-2.712752	USP17L29	ubiquitin	728405
ILMN_16737	8.71E-04	4.48E-05	1.1052351	KCNG1	potassium	3755
ILMN_17089	9.08E-04	4.71E-05	1.3322009	PDZD4	PDZ domain	57595
ILMN_20678	9.51E-04	4.98E-05	-1.109	SLC30A1	solute car	7779
ILMN_17984	9.60E-04	5.03E-05	-1.271562	KAZN	kazrin, pe	23254
ILMN_32349	1.11E-03	6.04E-05	-3.410533	SNORD105	small nucl	692229
ILMN_17829	9.80E-04	5.16E-05	1.426214	SLC16A10	solute car	117247
ILMN_23302	1.17E-03	6.41E-05	-2.288377	CUX1	cut like h	1523
ILMN_18050	1.03E-03	5.50E-05	-1.334823	PDE1B	phosphodie	5153
ILMN_17244	9.93E-04	5.25E-05	1.0411497	AXIN2	axin 2	8313
ILMN_16834	9.96E-04	5.27E-05	-1.230569	CDCA5	cell divis	113130
ILMN_22487	1.00E-03	5.33E-05	1.1115495	TYSND1	trypsin do	219743
ILMN_16514	1.15E-03	6.30E-05	-1.90372	LILRA6	leukocyte	79168
ILMN_23292	1.03E-03	5.51E-05	1.46162	TRIM10	tripartite	10107
ILMN_17923	1.04E-03	5.56E-05	1.1786604	RNF165	ring finge	494470
ILMN_17790	1.04E-03	5.61E-05	1.5588009	FEZ1	fasciculat	9638
ILMN_21982	1.07E-03	5.76E-05	1.0447089	HGD	homogentis	3081
ILMN_17533	1.08E-03	5.86E-05	-2.318388	DEFA4	defensin a	1669
ILMN_20476	1.39E-03	7.95E-05	-2.632178	KCNE1	potassium	3753
ILMN_21131	1.19E-03	6.53E-05	-1.614603	RNASE3	ribonuclea	6037
ILMN_17511	1.19E-03	6.57E-05	-1.12851	HIST1H4H	histone cl	8365
ILMN_17080	1.22E-03	6.77E-05	1.2335947	TMEM8B	transmembr	51754
ILMN_16694	1.22E-03	6.77E-05	1.0933721	PYHIN1	pyrin and	149628
ILMN_17433	1.22E-03	6.81E-05	1.3073482	DLL1	delta like	28514
ILMN_17103	1.26E-03	7.03E-05	1.7462481	TTC25	tetratrico	83538
ILMN_16748	1.25E-03	6.96E-05	-1.19735	OASL	2'-5'-olig	8638
ILMN_24075	1.27E-03	7.13E-05	-1.151902	RNF135	ring finge	84282
ILMN_17936	1.43E-03	8.26E-05	-1.522553	TUBA8	tubulin al	51807
ILMN_17417	1.57E-03	9.31E-05	-2.299598	MS4A4A	membrane s	51338
ILMN_32020	1.44E-03	8.33E-05	-2.021562	ECRP	ribonuclea	643332
ILMN_32351	1.51E-03	8.81E-05	-2.053443	BICDL2	BICD famil	146439

ILMN_17228	1.38E-03	7.83E-05	1.42289	NRCAM	neuronal c	4897
ILMN_17052	1.39E-03	7.98E-05	1.3329331	MYBPH	myosin bir	4608
ILMN_32449	1.45E-03	8.40E-05	-1.358139	SNORA78	small nucl	677844
ILMN_32489	1.40E-03	8.02E-05	1.1039762	MIR155HG	MIR155 hos	114614
ILMN_17005	1.48E-03	8.60E-05	-1.659969	C7orf34	chromosome	135927
ILMN_23952	1.41E-03	8.08E-05	1.1344272	FMNL3	formin lik	91010
ILMN_17639	1.42E-03	8.17E-05	-1.336394	CENPW	centromere	387103
ILMN_18480	1.44E-03	8.30E-05	1.3608035	IKZF2	IKAROS fam	22807
ILMN_21757	1.75E-03	1.06E-04	2.2398547	KIR2DS3	killer cel	3808
ILMN_22966	1.52E-03	8.92E-05	-1.135579	C9orf72	chromosome	203228
ILMN_16646	1.82E-03	1.11E-04	-2.047276	ARMCX3	armadillo	51566
ILMN_31813	1.49E-03	8.70E-05	-1.474333	MRVI1-AS1	MRVI1 anti	100129827
ILMN_17135	1.50E-03	8.73E-05	1.000039	LGR6	leucine ri	59352
ILMN_18146	1.51E-03	8.83E-05	1.1378428	ZC3H12B	zinc finge	340554
ILMN_16809	1.59E-03	9.44E-05	-2.031852	PTPN20	protein ty	26095
ILMN_17370	1.51E-03	8.84E-05	1.0583192	CAPN5	calpain 5	726
ILMN_24138	1.62E-03	9.64E-05	-1.323676	MCM10	minichromo	55388
ILMN_16892	1.61E-03	9.60E-05	1.1378207	SNORD14D	small nucl	85390
ILMN_17610	1.76E-03	1.06E-04	1.5460591	B3GAT1	beta-1,3-g	27087
ILMN_21296	1.70E-03	1.02E-04	-1.097927	RAB27B	RAB27B, me	5874
ILMN_17671	1.64E-03	9.79E-05	1.2627818	CAMK4	calcium/ca	814
ILMN_17588	1.66E-03	9.96E-05	-1.094813	ENTPD7	ectonucleo	57089
ILMN_17127	1.80E-03	1.09E-04	-1.45918	ZNF608	zinc finge	57507
ILMN_33084	2.03E-03	1.28E-04	-2.21752	MIR9-3	microRNA 9	407051
ILMN_16667	1.73E-03	1.04E-04	1.2885612	CXCL8	C-X-C moti	3576
ILMN_23291	1.74E-03	1.05E-04	1.1048497	COLQ	collagen l	8292
ILMN_17165	1.82E-03	1.11E-04	-1.435256	INTU	inturned p	27152
ILMN_17139	1.99E-03	1.24E-04	-1.828043	C1orf106	chromosome	55765
ILMN_17942	2.34E-03	1.51E-04	-2.663886	KCNJ12	potassium	3768
ILMN_16718	1.82E-03	1.11E-04	1.0938879	PID1	phosphotyr	55022
ILMN_16568	2.18E-03	1.39E-04	-2.134246	SLC26A8	solute car	116369
ILMN_24136	1.95E-03	1.21E-04	1.8381375	LLGL2	LLGL2, scr	3993
ILMN_17156	1.84E-03	1.13E-04	-1.073485	TP53I11	tumor prot	9537
ILMN_32458	1.88E-03	1.16E-04	1.3478146	SCARNA17	small Caja	677769
ILMN_17628	1.88E-03	1.16E-04	1.3837471	KLF12	Kruppel li	11278
ILMN_17736	2.12E-03	1.34E-04	-4.503684	OR7E91P	olfactory	79315
ILMN_16998	1.99E-03	1.24E-04	-1.007059	RAB27A	RAB27A, me	5873
ILMN_32351	1.94E-03	1.20E-04	1.0060588	TRABD2A	TraB domai	129293
ILMN_16925	1.98E-03	1.23E-04	1.0855668	VNN2	vanin 2	8875
ILMN_18088	2.00E-03	1.26E-04	1.3208894	NPR2	natriureti	4882
ILMN_23767	2.00E-03	1.25E-04	-1.241488	CDKN2B	cyclin dep	1030
ILMN_23031	1.97E-03	1.23E-04	1.2829808	MBNL3	muscleblin	55796
ILMN_16557	2.40E-03	1.56E-04	-2.829918	ANKRD34B	ankyrin re	340120
ILMN_17118	2.14E-03	1.36E-04	1.2361267	SLC24A2	solute car	25769
ILMN_23966	2.16E-03	1.38E-04	-1.290755	PDLIM7	PDZ and LI	9260
ILMN_16790	2.68E-03	1.78E-04	-2.264164	OR51A7	olfactory	119687
ILMN_18017	2.22E-03	1.42E-04	-1.114144	PSG9	pregnancy	5678

ILMN_1785§	2.67E-03	1.78E-04	-2.279392	C1QC	complement	714
ILMN_2256§	2.28E-03	1.47E-04	-1.195238	BTN2A2	butyrophil	10385
ILMN_1776§	2.30E-03	1.49E-04	1.2868457	MS4A1	membrane s	931
ILMN_2339∠	4.38E-04	1.96E-05	-7.498442	ACE	angiotensi	1636
ILMN_1733(	2.61E-03	1.72E-04	-1.900829	RAB36	RAB36, mem	9609
ILMN_2370:	2.59E-03	1.71E-04	-2.105323	MS4A4A	membrane s	51338
ILMN_1737§	2.36E-03	1.52E-04	1.0707483	ELOVL4	ELOVL fatt	6785
ILMN_1705§	2.38E-03	1.54E-04	-1.015744	HNMT	histamine	3176
ILMN_1692{	2.38E-03	1.54E-04	1.0271673	ITGB3BP	integrin s	23421
ILMN_1796§	2.39E-03	1.55E-04	-1.090711	TPX2	TPX2, micr	22974
ILMN_1729:	2.53E-03	1.66E-04	1.5127875	NMUR1	neuromedir	10316
ILMN_1731(	2.44E-03	1.59E-04	1.0546894	TMSB15B	thymosin b	286527
ILMN_1790(	2.43E-03	1.59E-04	1.1465484	GNLY	granulysir	10578
ILMN_2069:	2.45E-03	1.60E-04	1.6307719	PVALB	parvalbumi	5816
ILMN_1736§	2.49E-03	1.63E-04	-1.262548	PLB1	phospholip	151056
ILMN_1673{	2.81E-03	1.88E-04	-1.645854	EXO1	exonucleas	9156
ILMN_2240:	2.63E-03	1.75E-04	1.2476971	SYTL2	synaptotag	54843
ILMN_1652:	2.69E-03	1.79E-04	1.4193246	NOG	noggin	9241
ILMN_1733:	2.73E-03	1.83E-04	-1.205099	CD163	CD163 mole	9332
ILMN_3243§	3.16E-03	2.19E-04	-3.895671	HIST2H3A	histone cl	333932
ILMN_1806{	2.77E-03	1.85E-04	1.1342152	MS4A2	membrane s	2206
ILMN_1716]	2.81E-03	1.89E-04	1.1728817	FAXDC2	fatty acic	10826
ILMN_1739:	3.07E-03	2.11E-04	-1.717884	OMG	oligodendr	4974
ILMN_2384∠	2.82E-03	1.90E-04	1.1350809	RTBDN	retbindin	83546
ILMN_1708{	2.88E-03	1.94E-04	1.1923875	GNLY	granulysir	10578
ILMN_1741∠	2.94E-03	2.00E-04	-1.013264	USP30	ubiquitin	84749
ILMN_1661]	2.95E-03	2.00E-04	1.1476804	CLCF1	cardiotrop	23529
ILMN_1684:	3.03E-03	2.07E-04	1.3017727	MYL4	myosin lig	4635
ILMN_2411:	3.22E-03	2.23E-04	1.4709325	NRCAM	neuronal c	4897
ILMN_1689]	3.10E-03	2.13E-04	-1.03778	PLPP4	phospholip	196051
ILMN_1767]	3.24E-03	2.26E-04	-1.251831	CMIP	c-Maf indu	80790
ILMN_1826§	3.57E-03	2.55E-04	-1.931248	APBB2	amyloid be	323
ILMN_1790]	3.25E-03	2.27E-04	1.2674023	KIT	KIT proto-	3815
ILMN_2408]	3.27E-03	2.28E-04	1.1942519	CD200R1	CD200 rece	131450
ILMN_2353(	3.29E-03	2.29E-04	1.1520014	GRAP	GRB2-relat	10750
ILMN_3268∠	3.47E-03	2.45E-04	-1.174046	CCL14	C-C motif	6358
ILMN_2140:	3.43E-03	2.41E-04	1.1077615	DPH6	diphthamir	89978
ILMN_2371§	3.97E-03	2.92E-04	-2.133549	MUC1	mucin 1, c	4582
ILMN_2202§	3.67E-03	2.64E-04	-1.570304	BUB1	BUB1 mitot	699
ILMN_3243∠	3.47E-03	2.46E-04	-1.400796	LIPN	lipase fam	643418
ILMN_1707§	3.49E-03	2.47E-04	-1.651971	CARD17	caspase re	440068
ILMN_1756{	3.51E-03	2.49E-04	1.5826379	RUNDC3A	RUN domair	10900
ILMN_2376§	3.56E-03	2.53E-04	1.3537776	PDGFD	platelet c	80310
ILMN_2340§	3.87E-03	2.82E-04	-2.152197	GRB10	growth fac	2887
ILMN_1682§	3.59E-03	2.57E-04	-1.036116	PRKCD	protein ki	5580
ILMN_2349∠	3.67E-03	2.64E-04	-1.408163	BIRC5	baculovira	332
ILMN_1755∠	4.37E-03	3.28E-04	-1.699891	RBMS2	RNA bindir	5939

ILMN_1793[illegible]	3.71E-03	2.67E-04	1.3592814	MXI1	MAX intera	4601
ILMN_2201[illegible]	3.71E-03	2.68E-04	1.0147357	GSTM2	glutathior	2946
ILMN_1700[illegible]	3.89E-03	2.84E-04	1.0570864	UST	uronyl 2-s	10090
ILMN_1796[illegible]	3.97E-03	2.91E-04	-1.213897	TRIP13	thyroid hc	9319
ILMN_1754[illegible]	3.87E-03	2.82E-04	1.1507556	PASK	PAS domair	23178
ILMN_3293[illegible]	4.20E-03	3.13E-04	-1.039965	FAM27C	family wit	100132948
ILMN_1705[illegible]	4.01E-03	2.96E-04	-1.004658	ARHGEF40	Rho guanir	55701
ILMN_1731[illegible]	4.60E-03	3.50E-04	-1.544498	ORC6	origin rec	23594
ILMN_1663[illegible]	4.38E-03	3.29E-04	-2.701332	OTOF	otoferlin	9381
ILMN_1744[illegible]	5.49E-03	4.37E-04	-3.371839	KCNN3	potassium	3782
ILMN_1676[illegible]	4.30E-03	3.23E-04	1.2973827	SPON2	spondin 2	10417
ILMN_1676[illegible]	4.18E-03	3.11E-04	1.0287564	TCF7	transcript	6932
ILMN_1670[illegible]	4.24E-03	3.17E-04	-1.125488	WDFY3	WD repeat	23001
ILMN_1747[illegible]	4.25E-03	3.18E-04	-1.135261	CEP55	centrosoma	55165
ILMN_1679[illegible]	4.38E-03	3.30E-04	-1.307227	FAM110B	family wit	90362
ILMN_1693[illegible]	4.59E-03	3.49E-04	-1.441208	ASPH	aspartate	444
ILMN_1680[illegible]	4.25E-03	3.17E-04	-1.939086	CTSG	cathepsin	1511
ILMN_3309[illegible]	5.84E-03	4.73E-04	-2.093088	SNAR-I	small ILF[illegible]	100170222
ILMN_1760[illegible]	4.38E-03	3.30E-04	1.6754751	VWCE	von Willeb	220001
ILMN_1766[illegible]	4.96E-03	3.84E-04	-1.823696	PKMYT1	protein ki	9088
ILMN_2194[illegible]	4.55E-03	3.45E-04	1.4786756	PLVAP	plasmalemm	83483
ILMN_1774[illegible]	4.47E-03	3.38E-04	1.2489697	IL24	interleuki	11009
ILMN_2383[illegible]	4.46E-03	3.37E-04	1.0901735	SIRPG	signal reg	55423
ILMN_1737[illegible]	4.55E-03	3.45E-04	1.0650923	GGT7	gamma-glut	2686
ILMN_1739[illegible]	4.83E-03	3.71E-04	-1.228163	HIST1H2BO	histone cl	8348
ILMN_1676[illegible]	4.57E-03	3.47E-04	1.1318657	CABP5	calcium bi	56344
ILMN_2068[illegible]	5.36E-03	4.24E-04	-1.608332	AATK	apoptosis	9625
ILMN_1851[illegible]	5.27E-03	4.14E-04	-1.371036	FGF12	fibroblast	2257
ILMN_1725[illegible]	4.76E-03	3.65E-04	1.1021235	CARNS1	carnosine	57571
ILMN_1698[illegible]	5.10E-03	3.97E-04	-1.394667	FGD4	FYVE, Rho[illegible]	121512
ILMN_1728[illegible]	5.24E-03	4.11E-04	-1.31759	FAM64A	family wit	54478
ILMN_1665[illegible]	4.99E-03	3.88E-04	1.2812731	CLEC4C	C-type lec	170482
ILMN_1779[illegible]	4.99E-03	3.88E-04	-5.946034	C7orf33	chromosome	202865
ILMN_3235[illegible]	5.00E-03	3.88E-04	-1.38118	LOC644936	actin, bet	644936
ILMN_1684[illegible]	5.68E-03	4.55E-04	-1.834435	ARSD	arylsulfat	414
ILMN_3238[illegible]	2.16E-03	1.38E-04	-6.969123	LRRC70	leucine ri	100130733
ILMN_2160[illegible]	5.26E-03	4.12E-04	1.0080847	TMIGD2	transmembr	126259
ILMN_1724[illegible]	5.38E-03	4.26E-04	-1.001043	CARD16	caspase re	114769
ILMN_2335[illegible]	6.43E-03	5.32E-04	-1.96646	ITIH5	inter-alph	80760
ILMN_2325[illegible]	5.27E-03	4.13E-04	1.1385409	TYSND1	trypsin dc	219743
ILMN_1672[illegible]	6.07E-03	4.96E-04	-1.846048	OAS1	2'-5'-olig	4938
ILMN_1679[illegible]	7.00E-03	5.91E-04	-2.519726	ARSJ	arylsulfat	79642
ILMN_2059[illegible]	5.70E-03	4.58E-04	1.6144002	KLRC2	killer cel	3822
ILMN_1670[illegible]	5.72E-03	4.59E-04	-1.301157	CDC45	cell divis	8318
ILMN_3305[illegible]	5.87E-03	4.76E-04	-1.289042	ZNF788	zinc finge	388507
ILMN_3310[illegible]	7.59E-03	6.52E-04	-2.17482	MIR210	microRNA [illegible]	406992
ILMN_1676[illegible]	5.66E-03	4.52E-04	1.1829148	SIRPG	signal reg	55423

ILMN_2396[illegible]	5.66E-03	4.53E-04	-1.258717	RFX2	regulatory	5990
ILMN_1691[illegible]	5.71E-03	4.59E-04	-1.063194	SLC22A18AS	solute car	5003
ILMN_1804[illegible]	5.90E-03	4.79E-04	-1.346621	CBS	cystathior	875
ILMN_2296[illegible]	2.28E-03	1.47E-04	-7.488829	GH2	growth hor	2689
ILMN_2093[illegible]	5.79E-03	4.67E-04	1.1051103	ZNF571	zinc finge	51276
ILMN_2225[illegible]	6.30E-03	5.19E-04	-4.707634	TRIM71	tripartite	131405
ILMN_3243[illegible]	6.38E-03	5.27E-04	-1.555863	NRADDP	neurotroph	100129354
ILMN_1786[illegible]	5.82E-03	4.69E-04	-1.504402	LILRA3	leukocyte	11026
ILMN_1793[illegible]	8.24E-03	7.25E-04	-2.560434	OR2G3	olfactory	81469
ILMN_1766[illegible]	5.86E-03	4.75E-04	-1.040081	MRVI1	murine ret	10335
ILMN_1730[illegible]	7.66E-03	6.61E-04	-2.697046	TTC24	tetratrico	164118
ILMN_1724[illegible]	5.95E-03	4.84E-04	1.2904081	GCAT	glycine C-	23464
ILMN_1708[illegible]	6.07E-03	4.96E-04	-1.23951	NT5DC2	5'-nucleot	64943
ILMN_1663[illegible]	6.30E-03	5.19E-04	1.3619775	MTUS1	microtubul	57509
ILMN_1668[illegible]	7.45E-03	6.38E-04	-1.758137	AKR1C2	aldo-keto	1646
ILMN_1668[illegible]	8.35E-03	7.37E-04	-3.283744	RBPMS	RNA bindir	11030
ILMN_3308[illegible]	6.92E-03	5.83E-04	-5.190531	MIR590	microRNA 5	693175
ILMN_1751[illegible]	6.28E-03	5.17E-04	1.2973414	PACSIN1	protein ki	29993
ILMN_1801[illegible]	6.33E-03	5.23E-04	-1.219274	CCNB2	cyclin B2	9133
ILMN_1803[illegible]	8.18E-03	7.17E-04	-3.498639	BIRC5	baculovira	332
ILMN_3305[illegible]	8.47E-03	7.52E-04	-1.983393	LOC730159	uncharacte	730159
ILMN_1723[illegible]	6.63E-03	5.51E-04	-1.769054	PFKFB2	6-phosphof	5208
ILMN_3310[illegible]	8.90E-03	8.00E-04	-2.126286	MIR512-2	microRNA 5	574459
ILMN_1690[illegible]	7.56E-03	6.50E-04	-1.320041	AGER	advanced g	177
ILMN_1699[illegible]	6.72E-03	5.61E-04	1.0746048	CXCR5	C-X-C moti	643
ILMN_2188[illegible]	9.48E-03	8.66E-04	-2.792648	OR10H5	olfactory	284433
ILMN_2362[illegible]	8.21E-03	7.21E-04	-1.491183	ZWINT	ZW10 inter	11130
ILMN_1814[illegible]	6.78E-03	5.69E-04	1.4863343	EPB42	erythrocyt	2038
ILMN_3245[illegible]	8.90E-03	8.00E-04	-1.752584	LINC00895	long inter	150185
ILMN_3306[illegible]	8.20E-03	7.20E-04	-1.456291	C1QTNF9B-A	C1QTNF9B a	542767
ILMN_2092[illegible]	7.12E-03	6.03E-04	1.249376	BEND2	BEN domair	139105
ILMN_1654[illegible]	8.42E-03	7.45E-04	-1.295485	CCL18	C-C motif	6362
ILMN_1811[illegible]	7.09E-03	6.00E-04	1.1856828	OR2W3	olfactory	343171
ILMN_1654[illegible]	7.30E-03	6.20E-04	1.0703766	EFNB1	ephrin B1	1947
ILMN_1694[illegible]	7.34E-03	6.26E-04	1.518862	HMBS	hydroxymet	3145
ILMN_2409[illegible]	8.30E-03	7.32E-04	-1.505497	HMMR	hyaluronar	3161
ILMN_2173[illegible]	7.89E-03	6.86E-04	-1.083893	TBC1D3F	TBC1 domai	84218
ILMN_1816[illegible]	7.40E-03	6.32E-04	-1.085075	SLC8A1	solute car	6546
ILMN_1713[illegible]	7.43E-03	6.36E-04	1.0500597	GSTM2	glutathior	2946
ILMN_3237[illegible]	7.46E-03	6.40E-04	1.0955103	CLUHP3	clustered	100132341
ILMN_1746[illegible]	9.80E-03	9.01E-04	-3.822861	PCOLCE2	procollage	26577
ILMN_1656[illegible]	8.01E-03	6.99E-04	1.3228371	BNC2	basonuclir	54796
ILMN_1756[illegible]	8.07E-03	7.06E-04	-1.313103	MOV10L1	Mov10 RISC	54456
ILMN_1728[illegible]	7.63E-03	6.57E-04	-1.276112	FCGR3B	Fc fragmer	2215
ILMN_1742[illegible]	7.64E-03	6.58E-04	1.0196732	BCL2L1	BCL2 like	598
ILMN_1788[illegible]	8.66E-03	7.73E-04	-1.454481	KREMEN1	kringle cc	83999
ILMN_1656[illegible]	8.28E-03	7.29E-04	-1.412138	FBXO48	F-box prot	554251

ILMN_17351	7.72E-03	6.68E-04	1.0311207	GALNT12	polypeptic	79695
ILMN_18088	7.83E-03	6.81E-04	-1.090418	TBKBP1	TBK1 bindi	9755
ILMN_19036	8.15E-03	7.15E-04	-1.072617	LOC105376	uncharacte	105376544
ILMN_32355	8.12E-03	7.11E-04	1.2884525	GPR183	G protein-	1880
ILMN_23490	1.03E-02	9.62E-04	-2.190449	ADGRG2	adhesion (	10149
ILMN_17895	8.03E-03	7.02E-04	1.1135006	NBL1	neuroblast	4681
ILMN_21500	8.36E-03	7.39E-04	-1.356793	CES1P1	carboxyles	51716
ILMN_17255	8.36E-03	7.38E-04	-1.335166	DHCR24	24-dehydrc	1718
ILMN_17333	8.00E-03	6.98E-04	1.1072346	TEX9	testis exp	374618
ILMN_31883	8.80E-03	7.89E-04	-1.372441	HPN-AS1	HPN antise	100128675
ILMN_16590	8.07E-03	7.06E-04	1.1520202	CD40LG	CD40 ligar	959
ILMN_16513	8.66E-03	7.72E-04	1.4243594	SPP1	secreted p	6696
ILMN_22293	8.45E-03	7.48E-04	1.1391235	KIT	KIT proto-	3815
ILMN_16819	1.09E-02	1.03E-03	-1.946467	TMEM232	transmembr	642987
ILMN_16944	8.35E-03	7.37E-04	1.3974731	CRIP2	cysteine r	1397
ILMN_17986	8.38E-03	7.41E-04	1.0607522	ANO9	anoctamin	338440
ILMN_18125	8.68E-03	7.75E-04	1.3133543	RNF14	ring finge	9604
ILMN_23983	9.18E-03	8.32E-04	-1.192898	PML	promyelocy	5371
ILMN_16786	8.92E-03	8.03E-04	-1.236442	RRM2	ribonuclec	6241
ILMN_24150	1.18E-02	1.13E-03	-1.833947	ATP2B3	ATPase pla	492
ILMN_17026	1.13E-02	1.08E-03	-2.38527	B3GNT5	UDP-GlcNAc	84002
ILMN_17307	8.66E-03	7.71E-04	1.0773252	ADORA3	adenosine	140
ILMN_32705	9.01E-03	8.13E-04	1.3405881	KLF3-AS1	KLF3 antis	79667
ILMN_16533	1.15E-02	1.10E-03	-1.729847	IL22RA2	interleuki	116379
ILMN_17090	9.20E-03	8.34E-04	1.4985274	ABCC13	ATP bindir	150000
ILMN_17734	8.86E-03	7.96E-04	1.2191281	DOCK9	dedicator	23348
ILMN_22129	8.99E-03	8.11E-04	-1.326101	MELK	maternal e	9833
ILMN_17684	8.85E-03	7.95E-04	-1.176332	TCN1	transcobal	6947
ILMN_17949	1.21E-02	1.17E-03	-2.614888	ABCA4	ATP bindir	24
ILMN_16762	9.91E-03	9.14E-04	-1.242873	ABCC2	ATP bindir	1244
ILMN_17597	9.00E-03	8.11E-04	-1.139098	THBD	thrombomoc	7056
ILMN_17242	1.24E-02	1.21E-03	-2.376286	INSL6	insulin li	11172
ILMN_17220	9.02E-03	8.15E-04	-1.168256	ARSB	arylsulfat	411
ILMN_22256	9.54E-03	8.72E-04	1.1501318	FAM135A	family wit	57579
ILMN_16945	1.05E-02	9.85E-04	-1.734056	CLTCL1	clathrin h	8218
ILMN_16586	1.08E-02	1.01E-03	-1.381697	NUF2	NUF2, NDC8	83540
ILMN_17869	1.43E-02	1.45E-03	-2.775	OR52B2	olfactory	255725
ILMN_16580	1.29E-02	1.26E-03	2.1931341	ZNF365	zinc finge	22891
ILMN_17368	1.00E-02	9.24E-04	1.6878736	PRSS33	protease,	260429
ILMN_17162	1.43E-02	1.44E-03	-2.290951	PSG6	pregnancy	5675
ILMN_17919	1.01E-02	9.37E-04	-1.112901	RASGRP4	RAS guanyl	115727
ILMN_16922	9.80E-03	9.00E-04	-1.130038	LCN2	lipocalin	3934
ILMN_17079	9.83E-03	9.05E-04	-1.143755	ABHD12B	abhydrolas	145447
ILMN_23529	1.23E-02	1.19E-03	-1.814652	ASPH	aspartate	444
ILMN_16886	1.02E-02	9.46E-04	-1.011529	FBXL2	F-box and	25827
ILMN_17580	1.00E-02	9.27E-04	1.0128969	NFIA	nuclear fa	4774
ILMN_16678	1.45E-02	1.47E-03	2.1003451	XAGE5	X antigen	170627

ILMN_1676:	1.06E-02	9.97E-04	1.1891867	KDF1	keratinocy	126695
ILMN_2306E	1.01E-02	9.33E-04	1.0255663	PDE9A	phosphodie	5152
ILMN_1807	1.03E-02	9.64E-04	1.1254523	IKZF2	IKAROS fan	22807
ILMN_1769E	1.17E-02	1.12E-03	-1.41693	HORMAD1	HORMA doma	84072
ILMN_1663	1.07E-02	1.00E-03	1.0773862	LIN7B	lin-7 homc	64130
ILMN_1774	1.18E-02	1.14E-03	-1.280165	C2orf82	chromosome	389084
ILMN_1679	1.38E-02	1.39E-03	-2.268303	AP3B2	adaptor re	8120
ILMN_1660E	1.16E-02	1.11E-03	-1.58687	CDCA2	cell divis	157313
ILMN_1661	1.08E-02	1.02E-03	-1.08931	LILRA3	leukocyte	11026
ILMN_1802	1.09E-02	1.03E-03	-1.003768	FFAR3	free fatty	2865
ILMN_1792	1.20E-02	1.16E-03	-1.292706	SOD2	superoxide	6648
ILMN_1814	1.15E-02	1.10E-03	1.033386	TLE2	transducir	7089
ILMN_1812	1.27E-02	1.25E-03	-1.262194	ICA1	islet cell	3382
ILMN_3286	1.19E-02	1.14E-03	-1.052204	LOC284648	uncharacte	284648
ILMN_1668	1.20E-02	1.16E-03	1.0341769	BEGAIN	brain enri	57596
ILMN_1855	1.22E-02	1.18E-03	1.0582331	ADM5	adrenomedu	199800
ILMN_2381	1.18E-02	1.13E-03	-1.312539	DSC2	desmocolli	1824
ILMN_1761	1.53E-02	1.57E-03	-1.72142	C7orf57	chromosome	136288
ILMN_1730	1.29E-02	1.27E-03	-1.352821	VSIG8	V-set and	391123
ILMN_2141	1.21E-02	1.17E-03	1.2363263	SERPINF1	serpin fan	5176
ILMN_3245	1.21E-02	1.16E-03	-1.145311	CFAP45	cilia and	25790
ILMN_2058	1.21E-02	1.16E-03	-2.324563	IFI27	interferor	3429
ILMN_2109	1.22E-02	1.18E-03	1.0928944	GZMB	granzyme E	3002
ILMN_1815	8.06E-03	7.05E-04	-6.477313	NR5A1	nuclear re	2516
ILMN_1813	1.31E-02	1.29E-03	1.245589	RORA	RAR relate	6095
ILMN_2129	1.68E-02	1.76E-03	-2.024653	LDLRAD3	low densit	143458
ILMN_1696	1.79E-02	1.91E-03	-2.103812	ANKS1B	ankyrin re	56899
ILMN_1651	1.49E-02	1.52E-03	-1.329224	RTN3	reticulon	10313
ILMN_1701	1.32E-02	1.30E-03	1.0759091	STRADB	STE20-rela	55437
ILMN_3238	1.49E-02	1.52E-03	-1.95328	CARD17	caspase re	440068
ILMN_1760	1.40E-02	1.40E-03	1.217061	SHISA2	shisa fami	387914
ILMN_2375	2.05E-02	2.26E-03	-2.839182	COL4A5	collagen t	1287
ILMN_1779	1.45E-02	1.46E-03	1.2217361	CDH2	cadherin 2	1000
ILMN_2102	1.38E-02	1.39E-03	1.3110269	GATA2	GATA bindi	2624
ILMN_1865	1.41E-02	1.42E-03	1.1413249	LOC729451	uncharacte	729451
ILMN_2233	2.20E-02	2.46E-03	-2.356461	MYH16	myosin hea	84176
ILMN_2388	1.42E-02	1.44E-03	-1.080277	EPSTI1	epithelial	94240
ILMN_1797	1.55E-02	1.60E-03	1.202842	TGM2	transgluta	7052
ILMN_1694	1.45E-02	1.47E-03	1.0954534	SESN3	sestrin 3	143686
ILMN_1746	1.57E-02	1.62E-03	-1.125343	DYNAP	dynactin a	284254
ILMN_1776	1.53E-02	1.56E-03	-1.033173	C17orf53	chromosome	78995
ILMN_2329	1.70E-02	1.79E-03	-1.412521	CEP170	centrosoma	9859
ILMN_2086	2.02E-02	2.21E-03	-2.958419	PDGFRA	platelet c	5156
ILMN_1657	1.53E-02	1.57E-03	1.0253353	SIRT4	sirtuin 4	23409
ILMN_1735	2.07E-02	2.28E-03	-4.629036	FBXW11	F-box and	23291
ILMN_1659	1.69E-02	1.78E-03	-1.218563	KCNJ15	potassium	3772
ILMN_1667	1.62E-02	1.68E-03	1.3386814	NKX3-1	NK3 homeot	4824

ILMN_1789[illegible]	1.74E-02	1.84E-03	-1.527537	PKD2L1	polycystir	9033
ILMN_2172[illegible]	2.05E-02	2.25E-03	-1.33429	SLC18A1	solute car	6570
ILMN_1791[illegible]	1.59E-02	1.66E-03	1.1094669	AHI1	Abelson he	54806
ILMN_1813[illegible]	1.71E-02	1.81E-03	-1.306569	NEK11	NIMA relat	79858
ILMN_1733[illegible]	1.59E-02	1.65E-03	1.0535383	KIR3DL1	killer cel	3811
ILMN_2281[illegible]	2.01E-02	2.20E-03	-1.727519	PSG4	pregnancy	5672
ILMN_1781[illegible]	1.62E-02	1.69E-03	-1.060236	HMMR	hyaluronar	3161
ILMN_2144[illegible]	1.70E-02	1.79E-03	1.1764577	GOLGA6B	golgin A6	55889
ILMN_1785[illegible]	1.72E-02	1.82E-03	-1.011346	ERMAP	erythrobla	114625
ILMN_2068[illegible]	1.75E-02	1.86E-03	1.0957914	CLEC9A	C-type lec	283420
ILMN_1723[illegible]	1.73E-02	1.83E-03	-1.73206	OLR1	oxidized l	4973
ILMN_1760[illegible]	2.67E-02	3.16E-03	-2.202799	KIRREL2	kin of IRI	84063
ILMN_3237[illegible]	1.71E-02	1.80E-03	1.0593817	BEND2	BEN domair	139105
ILMN_1815[illegible]	2.22E-02	2.49E-03	1.5054232	DKK3	dickkopf W	27122
ILMN_1795[illegible]	1.89E-02	2.05E-03	-1.426029	GPER1	G protein-	2852
ILMN_3251[illegible]	1.77E-02	1.89E-03	-1.030876	LDHA	lactate de	3939
ILMN_2165[illegible]	1.80E-02	1.93E-03	1.6294962	ITLN1	intelectir	55600
ILMN_1728[illegible]	1.97E-02	2.15E-03	-1.303977	S100A16	S100 calci	140576
ILMN_1890[illegible]	1.79E-02	1.91E-03	1.1959309	TRAV20	T cell rec	28663
ILMN_3200[illegible]	2.43E-02	2.81E-03	-2.040047	ARMCX4	armadillo	100131755
ILMN_2042[illegible]	1.87E-02	2.02E-03	-1.17096	MRPL42P5	mitochondr	359821
ILMN_1809[illegible]	1.53E-02	1.56E-03	-5.719719	CADPS	calcium de	8618
ILMN_1810[illegible]	2.03E-02	2.23E-03	-1.506823	IL1R1	interleuki	3554
ILMN_1768[illegible]	2.62E-02	3.08E-03	-2.275875	TSN	translin	7247
ILMN_1766[illegible]	2.26E-02	2.56E-03	-1.203973	RBMS1	RNA bindir	5937
ILMN_3245[illegible]	2.72E-02	3.23E-03	-2.668831	PWAR1	Prader Wil	145624
ILMN_2184[illegible]	2.26E-02	2.56E-03	-1.990266	OAS3	2'-5'-olig	4940
ILMN_1806[illegible]	1.91E-02	2.08E-03	1.051826	PDE7B	phosphodie	27115
ILMN_3251[illegible]	2.34E-02	2.69E-03	-1.715908	NBPF7	neuroblast	343505
ILMN_1709[illegible]	2.79E-02	3.34E-03	-2.431873	C6orf10	chromosome	10665
ILMN_1796[illegible]	1.69E-02	1.77E-03	-5.366726	SLC13A3	solute car	64849
ILMN_2163[illegible]	2.93E-02	3.54E-03	-2.724931	CGB5	chorionic	93659
ILMN_2259[illegible]	2.26E-02	2.55E-03	-1.409971	BCKDHB	branched c	594
ILMN_1773[illegible]	2.98E-02	3.63E-03	-2.150429	XCR1	X-C motif	2829
ILMN_1802[illegible]	2.81E-02	3.37E-03	-3.698489	TAS2R50	taste 2 re	259296
ILMN_3309[illegible]	2.89E-02	3.49E-03	-2.276299	MIR30E	microRNA 3	407034
ILMN_1673[illegible]	1.95E-02	2.12E-03	1.026707	MAT2B	methionine	27430
ILMN_1653[illegible]	2.30E-02	2.61E-03	-1.328874	LAMC2	laminin su	3918
ILMN_1724[illegible]	2.41E-02	2.78E-03	-1.765426	OR10T2	olfactory	128360
ILMN_1794[illegible]	2.22E-02	2.49E-03	-1.377372	ZKSCAN7	zinc finge	55888
ILMN_1744[illegible]	2.51E-02	2.93E-03	-1.639064	ARHGAP11A	Rho GTPase	9824
ILMN_3243[illegible]	2.10E-02	2.32E-03	1.105545	SNORA17B	small nucl	677824
ILMN_3309[illegible]	2.72E-02	3.23E-03	-1.567269	MIR543	microRNA 5	100126335
ILMN_2287[illegible]	2.33E-02	2.66E-03	-1.354023	HIF3A	hypoxia ir	64344
ILMN_1757[illegible]	2.15E-02	2.39E-03	-1.236045	UCHL1	ubiquitin	7345
ILMN_1742[illegible]	3.14E-02	3.88E-03	-2.659593	ADCY1	adenylate	107
ILMN_1687[illegible]	2.11E-02	2.34E-03	-1.212728	GAS7	growth arr	8522

ILMN_2311(	2.06E-02	2.27E-03	-1.03824	BRCA1	BRCA1, DN/	672
ILMN_2145(	2.55E-02	2.99E-03	-2.281171	CCR5	C-C motif	1234
ILMN_2135:	2.85E-02	3.43E-03	-1.70961	CHDH	choline de	55349
ILMN_2293'	3.01E-02	3.68E-03	-2.257059	MARVELD2	MARVEL don	153562
ILMN_1725(	2.23E-02	2.50E-03	-1.326968	DPY19L3	dpy-19 lik	147991
ILMN_1705:	2.79E-02	3.32E-03	-2.361743	SPAG1	sperm ass(	6674
ILMN_1788:	2.14E-02	2.38E-03	1.172028	TOX	thymocyte	9760
ILMN_1682:	2.16E-02	2.41E-03	1.0890425	CLEC4C	C-type lec	170482
ILMN_1669:	3.15E-02	3.90E-03	-3.104678	PCDHA10	protocadhe	56139
ILMN_1761:	2.46E-02	2.86E-03	-1.34597	CACNA1A	calcium vo	773
ILMN_1731:	3.31E-02	4.14E-03	-3.673221	NXF2B	nuclear RI	728343
ILMN_3269]	3.47E-02	4.40E-03	-3.008794	LOC100129:	beta-defer	100129216
ILMN_1790'	2.57E-02	3.01E-03	-1.322573	C8orf31	chromosome	286122
ILMN_2211(	2.41E-02	2.78E-03	-1.292038	TTTY6	testis-spe	84672
ILMN_2184:	2.24E-02	2.52E-03	1.3212643	CXCL8	C-X-C moti	3576
ILMN_1688:	2.52E-02	2.94E-03	1.0388734	CELA2B	chymotryps	51032
ILMN_1754:	3.55E-02	4.53E-03	-3.759811	RGS9	regulator	8787
ILMN_2066(	2.26E-02	2.56E-03	1.861417	HLA-DRB6	major hist	3128
ILMN_1748'	2.93E-02	3.54E-03	-1.548192	SEC16B	SEC16 homo	89866
ILMN_1676:	3.16E-02	3.91E-03	-2.277167	HTRA1	HtrA serir	5654
ILMN_1670(	2.33E-02	2.66E-03	1.0594461	LPAR4	lysophosph	2846
ILMN_1749]	3.44E-02	4.35E-03	-4.225615	RXFP1	relaxin/ir	59350
ILMN_3242(	3.52E-02	4.49E-03	-2.871695	GTF2H2C_2	GTF2H2 fam	730394
ILMN_1771]	2.33E-02	2.67E-03	-1.018656	TMEM45B	transmembr	120224
ILMN_1702'	3.54E-02	4.51E-03	-2.757389	PGM5P2	phosphoglu	595135
ILMN_1801:	2.38E-02	2.73E-03	-1.203948	CENPA	centromere	1058
ILMN_2157:	3.17E-02	3.93E-03	-1.550859	MNS1	meiosis sp	55329
ILMN_1672:	3.12E-02	3.85E-03	-1.721831	TRIM55	tripartite	84675
ILMN_2150]	4.09E-02	5.42E-03	-2.371783	ZRANB3	zinc finge	84083
ILMN_1793:	2.55E-02	2.98E-03	-1.210122	RPTOR	regulatory	57521
ILMN_1712:	2.82E-02	3.38E-03	-1.61317	FAM89A	family wit	375061
ILMN_1691:	3.39E-02	4.27E-03	-1.785946	OR4C16	olfactory	219428
ILMN_3308(	3.14E-02	3.87E-03	-1.710161	MIR517B	microRNA 5	574483
ILMN_1686(	2.43E-02	2.81E-03	-1.055802	TOP2A	topoisomer	7153
ILMN_1770:	2.44E-02	2.82E-03	1.1017809	TMPRSS9	transmembr	360200
ILMN_1800:	3.98E-02	5.24E-03	-3.832327	WNT5A	Wnt family	7474
ILMN_1663:	2.46E-02	2.85E-03	-1.02379	CDC20	cell divis	991
ILMN_1798:	2.74E-02	3.26E-03	1.2699284	KRTAP11-1	keratin as	337880
ILMN_1758:	3.80E-02	4.96E-03	-2.415154	ANGPT4	angiopoiet	51378
ILMN_1724:	2.52E-02	2.94E-03	1.0545601	LYPD2	LY6/PLAUR	137797
ILMN_2294:	2.89E-02	3.48E-03	-1.189532	CTDP1	CTD phosph	9150
ILMN_1761:	3.89E-02	5.09E-03	-2.435371	FHOD3	formin hom	80206
ILMN_2085:	3.32E-02	4.16E-03	-1.780607	SNX24	sorting ne	28966
ILMN_1758(	3.93E-02	5.16E-03	-1.965014	CCNY	cyclin Y	219771
ILMN_2105:	2.65E-02	3.13E-03	1.1206644	TMEM220	transmembr	388335
ILMN_1684:	2.66E-02	3.14E-03	-1.053529	AURKB	aurora kir	9212
ILMN_2051:	2.72E-02	3.23E-03	-1.10038	DPRXP4	divergent-	503645

ILMN_1730З	3.77E-02	4.89E-03	-3.016086	CASP7	caspase 7	840
ILMN_22956	4.01E-02	5.29E-03	-2.24831	MAZ	MYC associ	4150
ILMN_17936	2.92E-02	3.52E-03	1.441341	ME3	malic enzy	10873
ILMN_16695	4.10E-02	5.44E-03	-2.838036	BZW1	basic leuc	9689
ILMN_17856	2.81E-02	3.36E-03	1.5653874	PMP22	peripheral	5376
ILMN_16711	2.63E-02	3.10E-03	1.2023168	GPR68	G protein-	8111
ILMN_17275	4.08E-02	5.41E-03	-4.049797	SULT2B1	sulfotrans	6820
ILMN_17126	4.04E-02	5.34E-03	1.4171918	ZNF829	zinc finge	374899
ILMN_17449	2.80E-02	3.34E-03	1.1197532	PTPRM	protein ty	5797
ILMN_20626	2.78E-02	3.32E-03	1.0718493	NMT2	N-myristoy	9397
ILMN_16981	2.78E-02	3.32E-03	-1.281863	INCENP	inner cent	3619
ILMN_32464	2.74E-02	3.26E-03	1.0476927	LOC1001308	uncharacte	100130872
ILMN_32399	2.73E-02	3.25E-03	1.5411731	IDO1	indoleamir	3620
ILMN_17089	2.79E-02	3.33E-03	1.0317711	TMEM161A	transmembr	54929
ILMN_16576	4.28E-02	5.74E-03	-3.797678	ZFHX4	zinc finge	79776
ILMN_17155	3.00E-02	3.66E-03	-1.013885	ZC3H11A	zinc finge	9877
ILMN_23852	2.96E-02	3.58E-03	-1.26195	PFKFB2	6-phosphof	5208
ILMN_17785	2.97E-02	3.60E-03	1.1330221	WNT7A	Wnt family	7476
ILMN_17195	3.97E-02	5.23E-03	-2.38197	C10orf67	chromosome	256815
ILMN_20543	3.44E-02	4.35E-03	-1.472229	SMIM21	small inte	284274
ILMN_16543	2.82E-02	3.38E-03	1.012705	HAPLN3	hyaluronar	145864
ILMN_17651	4.26E-02	5.72E-03	-3.488454	CST8	cystatin 8	10047
ILMN_17810	4.28E-02	5.74E-03	-1.923888	TSNARE1	t-SNARE dc	203062
ILMN_23945	4.28E-02	5.75E-03	-4.035499	WNK3	WNK lysine	65267
ILMN_17648	4.34E-02	5.85E-03	-3.190675	EML1	echinoderm	2009
ILMN_17916	3.01E-02	3.68E-03	-1.004184	KIAA0825	KIAA0825	285600
ILMN_16746	2.99E-02	3.64E-03	-1.55854	OLAH	oleoyl-ACF	55301
ILMN_17355	4.47E-02	6.06E-03	-3.015798	PKD1L2	polycystir	114780
ILMN_23471	3.79E-02	4.93E-03	1.4557798	DCN	decorin	1634
ILMN_32146	2.98E-02	3.63E-03	1.1983562	ZCCHC18	zinc finge	644353
ILMN_33096	4.62E-02	6.32E-03	-1.983769	MIR181A2	microRNA 1	406954
ILMN_20533	4.60E-02	6.28E-03	-3.689868	KCNT2	potassium	343450
ILMN_32460	3.28E-02	4.09E-03	-1.170906	NCMAP	non-compac	400746
ILMN_16708	3.30E-02	4.13E-03	-1.074246	ASB17	ankyrin re	127247
ILMN_23391	4.72E-02	6.49E-03	-1.764315	HMGCLL1	3-hydroxym	54511
ILMN_17511	3.45E-02	4.36E-03	-4.974752	CLDN14	claudin 14	23562
ILMN_16529	3.40E-02	4.29E-03	-1.020204	CLDN25	claudin 25	644672
ILMN_32445	4.66E-02	6.38E-03	-1.772642	ZSCAN23	zinc finge	222696
ILMN_16837	3.73E-02	4.82E-03	-1.419627	LHX6	LIM homeob	26468
ILMN_16805	3.18E-02	3.93E-03	1.0320366	RTKN	rhotekin	6242
ILMN_17343	3.25E-02	4.05E-03	1.0621624	RORC	RAR relate	6097
ILMN_18091	3.17E-02	3.92E-03	1.0889088	FAM118A	family wit	55007
ILMN_17311	3.48E-02	4.42E-03	-1.237954	CR1	complement	1378
ILMN_17187	4.50E-02	6.13E-03	-1.55885	XCL1	X-C motif	6375
ILMN_17627	3.22E-02	4.01E-03	1.0984544	MAGED1	MAGE famil	9500
ILMN_16902	3.20E-02	3.97E-03	-1.338807	BATF2	basic leuc	116071
ILMN_16529	3.54E-02	4.52E-03	-1.35	NPIPB3	nuclear pc	23117

ILMN_1711(	3.89E-02	5.09E-03	-1.474844	KCNB1	potassium	3745
ILMN_1748:	3.55E-02	4.54E-03	-1.047293	GGT8P	gamma-glut	645367
ILMN_1730£	4.51E-02	6.14E-03	-2.027604	HLA-DPB2	major hist	3116
ILMN_32397	3.25E-02	4.05E-03	-1.021489	DLGAP5	DLG associ	9787
ILMN_18117	3.68E-02	4.75E-03	-1.198311	MYO7A	myosin VII	4647
ILMN_1657:	4.24E-02	5.68E-03	-1.371519	EPHA6	EPH recept	285220
ILMN_2117£	4.74E-02	6.53E-03	-1.352755	DPPA3	developmer	359787
ILMN_22977	3.29E-02	4.11E-03	-1.082822	KCNMA1	potassium	3778
ILMN_3308(	3.88E-02	5.07E-03	1.3675723	MIR532	microRNA 5	693124
ILMN_1692(	4.96E-02	6.91E-03	-2.165258	FMNL3	formin lik	91010
ILMN_21861	3.38E-02	4.26E-03	1.1272454	DGCR6	DiGeorge s	8214
ILMN_2051:	3.87E-02	5.05E-03	-1.163416	NEK2	NIMA relat	4751
ILMN_1682(	3.51E-02	4.47E-03	-1.211873	TNFAIP8L3	TNF alpha	388121
ILMN_1675£	4.76E-02	6.56E-03	-1.524269	PRM1	protamine	5619
ILMN_1666{	3.55E-02	4.54E-03	-1.020367	DACH1	dachshund	1602
ILMN_23687	4.28E-02	5.74E-03	-1.924309	CENPM	centromere	79019
ILMN_1815(	3.67E-02	4.73E-03	1.2634739	PDGFRB	platelet c	5159
ILMN_17167	3.49E-02	4.43E-03	1.083621	MYOM2	myomesin 2	9172
ILMN_1760(	3.55E-02	4.54E-03	-1.03682	IFI44	interferon	10561
ILMN_2282:	4.72E-02	6.49E-03	1.2911114	MLH3	mutL homol	27030
ILMN_17777	3.69E-02	4.77E-03	1.1938843	FIGNL2	fidgetin l	401720
ILMN_1703	4.11E-02	5.46E-03	1.1270164	ATP5I	ATP syntha	521
ILMN_2210(	4.69E-02	6.44E-03	-4.724549	DNAJC25-GN	DNAJC25-GN	552891
ILMN_1685(	4.72E-02	6.49E-03	1.5023562	FLRT1	fibronecti	23769
ILMN_1690:	4.15E-02	5.52E-03	-1.285333	BFSP2	beaded fil	8419
ILMN_1657(	4.72E-02	6.50E-03	-1.271891	B3GALNT1	beta-1,3-N	8706
ILMN_32421	4.88E-02	6.77E-03	2.0706631	KIR3DS1	killer cel	3813
ILMN_17291	4.29E-02	5.76E-03	1.1445509	GATM	glycine am	2628
ILMN_1775{	4.87E-02	6.75E-03	-1.445841	TM4SF20	transmembr	79853
ILMN_16767	4.11E-02	5.46E-03	1.0270606	WDR33	WD repeat	55339
ILMN_1653(	4.20E-02	5.60E-03	-1.091963	HSD3B7	hydroxy-de	80270
ILMN_1722	4.50E-02	6.11E-03	-1.260081	HRH2	histamine	3274
ILMN_1756(	4.57E-02	6.22E-03	-1.576798	GBP6	guanylate	163351
ILMN_16637	4.04E-02	5.35E-03	1.0440636	EPB41	erythrocyt	2035
ILMN_1714(	4.89E-02	6.79E-03	-1.465208	SLC9A5	solute car	6553
ILMN_1876	4.85E-02	6.72E-03	-1.476681	MMP8	matrix met	4317
ILMN_1675(	4.60E-02	6.29E-03	-1.10853	ANKRD9	ankyrin re	122416
ILMN_17901	4.83E-02	6.68E-03	-1.092688	HGF	hepatocyte	3082
ILMN_1757£	4.44E-02	6.01E-03	1.0121646	USP40	ubiquitin	55230
ILMN_2082£	4.49E-02	6.10E-03	1.2052853	KIR3DL3	killer cel	115653
ILMN_1733:	4.81E-02	6.64E-03	-1.007013	TMIGD3	transmembr	57413
